# Network meta-analysis: relative clinical efficacy and safety of elafibranor versus seladelpar as second-line treatment for patients with primary biliary cholangitis

**DOI:** 10.57264/cer-2025-0206

**Published:** 2026-04-17

**Authors:** David Jones, Emily Combe, Harun Knight, Vicki Laskier-Owens, Shijie Ren, Tom Wright, Elaine A Böing, Alex Pashley

**Affiliations:** 1 Institute of Cellular Medicine & NIHR Newcastle Biomedical Research Centre, Newcastle University, Newcastle Upon Tyne, UK; 2 FIECON, London, UK; 3 Sheffield Centre for Health & Related Research, University of Sheffield, Sheffield, UK; 4 Ipsen, Cambridge, MA 02142, USA; 5 Costello Medical, Cambridge, UK

**Keywords:** autoimmune liver disease, liver, meta-analyses, outcomes research, primary biliary cholangitis

## Abstract

**Aim:**

To indirectly compare the efficacy and safety of elafibranor and seladelpar, as second-line treatments for primary biliary cholangitis.

**Materials & methods:**

Bayesian network meta-analyses compared data from randomized-controlled studies of elafibranor and seladelpar identified by a systematic literature review up to June 2024: (a) elafibranor (n = 108) versus placebo (n = 53; ELATIVE [NCT04526665]) and (b) seladelpar (n = 128) versus placebo (n = 65; RESPONSE [NCT03301506]). Patients from ELATIVE not meeting the RESPONSE upper limit of normal (ULN) criteria for alkaline phosphatase (ALP) and total bilirubin were excluded (n = 16); summary statistics for ELATIVE were recalculated using the new dataset. Random-effects models assessed the outcomes of cholestasis response (ALP <1.67 × ULN, ALP reduction ≥15% from baseline and total bilirubin ≤ULN), ALP normalization, change from baseline in ALP and pruritus, pruritus as a treatment-emergent adverse event and all-cause discontinuation.

**Results:**

Elafibranor-treated patients had greater odds of achieving cholestasis response than placebo- (median odds ratio [95% credible interval]: 84.79 [12.49, 2513.00]) or seladelpar-treated patients (13.02 [1.45, 420.20]), with posterior probabilities ≥99% that odds were higher with elafibranor than seladelpar or placebo. Among patients with ALP ≥350 U/l, the median odds ratio [95% credible interval] of cholestasis response for elafibranor-treated patients versus seladelpar-treated patients was 18.71 [0.65, 10,610.00], with a 95.2% posterior probability that odds were higher with elafibranor than seladelpar. For all other outcomes, there was no strong evidence of a difference between treatments.

**Conclusion:**

Bayesian network meta-analyses found strong probabilistic evidence supporting the treatment benefit of elafibranor compared with seladelpar for the achievement of cholestasis response at 52 weeks, while the treatment effect on other outcomes was uncertain. Head-to-head studies are needed to validate results of these indirect comparisons.

## Plain language summary: Comparison of elafibranor & seladelpar treatment in patients with primary biliary cholangitis following previous treatment with ursodeoxycholic acid

**What is this article about?:**

Primary biliary cholangitis (PBC) is a chronic disease that gradually damages the liver over time. This damage can lead to a build-up of symptoms and life-threatening complications, such as scarring (cirrhosis) and failure of the liver. Up to 40% of people living with PBC incompletely respond to first-line treatment with ursodeoxycholic acid. Recently, two new second-line treatments for PBC were launched, called elafibranor and seladelpar, which have similar ways of working. At present, there are no head-to-head trials of elafibranor and seladelpar, and we are not aware of any planned trials. Thus, this study aimed to compare the effectiveness of the two second-line treatments using a technique called an ‘indirect treatment comparison’. The objective is to help doctors and patients decide which of these treatments might be more suitable for different people. The approach compared the likelihood of clinical events from data provided across individual studies of elafibranor and seladelpar.

**What were the results?:**

We find that patients treated with elafibranor had a significantly higher chance of improving liver blood tests (specifically, cholestasis response) compared with those on seladelpar. However, for other outcomes, there was no significant difference between the treatments.

**Why is this important?:**

These findings could support treatment decisions for patients with PBC who respond poorly to ursodeoxycholic acid.

Patients with primary biliary cholangitis (PBC) experience a substantial clinical burden, with disease progression leading to accumulation of symptoms, comorbidities and life-threatening liver-related complications [1–3]. While most patients are asymptomatic at presentation [4,5], up to 70% and 80% of patients develop itching (pruritus) and fatigue, respectively [5–7], significantly impacting health-related quality-of-life. As PBC advances, the risk of complications rise, consequently necessitating transplantation in end-stage liver disease [8]. Indications for liver transplantation vary between countries, but broadly include hepatocellular carcinoma, persistent jaundice, hepatic decompensation and/or pharmacotherapy-refractory pruritus [9].

PBC is more therapy-responsive in its early stages [8]. Notably, alkaline phosphatase (ALP) and total bilirubin (TB) values under ursodeoxycholic acid (UDCA) treatment are predictive of liver-related events, and widely accepted biochemical surrogates of long-term outcomes [10,11]. This has been extrapolated in the development of second-line therapy, wherein biochemical cholestasis response (defined as an ALP <1.67 × upper limit of normal [ULN], TB≤ULN and ALP decrease from baseline of ≥15%) has been adopted as a primary efficacy outcome measure in clinical trials [12–14].

Multicenter and population-based data indicate that ∼40% of patients inadequately respond to UDCA, with 3–5% of patients being UCDA-intolerant [10,15,16], highlighting a need for effective second-line treatments. Elafibranor and seladelpar have recently been approved by the US FDA, EMA and Medicines and Healthcare products Regulatory Agency in the UK [17–22], providing new second-line treatment options for patients who inadequately respond to, or who are UDCA-intolerant.

Elafibranor, a peroxisome proliferator-activated receptor (PPAR) agonist exerting effects on both α and δ isoforms, has demonstrated efficacy in multicenter randomized controlled trials (RCTs) [13,23]. Notably, significantly more elafibranor-treated patients achieved cholestasis response at 52 weeks than placebo-treated patients in the Phase III ELATIVE RCT [13]. Significant differences between elafibranor- and placebo-treated patients were also observed for ALP normalization. Similarly, seladelpar, a selective PPAR-δ agonist, was evaluated in the Phase III RESPONSE RCT, wherein significantly more seladelpar-treated patients achieved cholestasis response at 52 weeks than placebo-treated patients [14]. ALP normalization was also achieved by significantly more seladelpar-treated patients than placebo-treated patients. However, comparing efficacy and safety across these trials is challenging due to different definitions of ULN for ALP and TB in trial eligibility criteria and response outcomes.

The lack of head-to-head RCTs comparing elafibranor and seladelpar poses challenges for decision-makers in evaluating their relative efficacy and safety. Consequently, using indirect treatment comparison (ITC) methods and existing clinical trial data, this study compares the relative efficacy and safety of elafibranor and seladelpar in adult patients with PBC who have an inadequate response or intolerance to UDCA.

## Materials & methods

### Study selection

A systematic literature review (SLR) was conducted to identify evidence describing the clinical efficacy and/or safety of investigational treatments for adult patients with PBC. The SLR was conducted in accordance with the methodological principles to conduct SLRs recommended by the Centre for Reviews and Dissemination (CRD), and with a prespecified protocol registered on PROSPERO (ID: CRD42023382262) [24].

The eligibility criteria were developed using the Population, Intervention, Comparator, Outcomes, Study design (PICOS) framework (Supplementary Table 1). Eligible studies investigated unselected adult patients with PBC who received monotherapy or combination therapy in any treatment line including, but not limited to elafibranor, UDCA, obeticholic acid, fibrates and seladelpar. Clinical studies that reported clinical effectiveness, pruritus, or safety outcomes were eligible for inclusion. Comprehensive search strategies were defined according to the PICOS framework, combining subject headings and test words for PBC, plus terms for interventional and observational studies based on validated search filters (Supplementary Tables 2 & 3). Searches were conducted within Medline, EMBASE, Cochrane Central Register of Controlled Trials, Cochrane Database of Systematic Reviews and the Database of Abstracts of Reviews of Effects from their inception to identify peer-reviewed publications in English. Database searches were conducted up to 19 June 2024. Conference proceedings for the American Association for the Study of Liver Diseases Liver Meeting, European Association for the Study of the Liver International Liver Congress, European Association for the Study of the Liver Digital Liver Cancer Summit, International Conference on Hepatology and Liver Disease and World Conference on Gastroenterology and Hepatology from 2021 to 2024 were last searched on 22 July 2024 (Supplementary Tables 4–6). Clinical trials registry searches were also last performed on 12 July 2024 in ClinicalTrials.gov (Supplementary Table 7). Further details on the dates of searches for the SLR are included in Section S1 of the Supplementary materials. The bibliographies of relevant SLRs and network meta-analyses (NMAs) identified during the SLR were also hand-searched to identify any additional, relevant studies for inclusion.

Studies were screened by two independent reviewers (AP, SS, EW; see Acknowledgements) for inclusion against the predefined eligibility criteria in two stages: first, the titles and abstracts of the search results; second, the full texts of potentially relevant articles, in order to obtain the final list of included studies. Disagreements were adjudicated by a third reviewer to arbitrate any discrepancies.

Due to the high volume of evidence identified in the SLR, a two-stage prioritization strategy was implemented. Interventional and observational studies in the second-line or later settings were prioritized following the title/abstract review phase. Subsequently, any studies of elafibranor and RCTs in the second-line or later setting were prioritized for extraction. Data extraction were performed by a single reviewer and verified independently by a second reviewer (AP, SS, EW; see Acknowledgements).

### Feasibility assessment

As the objective of the ITC was to compare elafibranor and seladelpar for second-line treatment of PBC, only studies including elafibranor and seladelpar were retained. Eligibility criteria for the ITC, developed using the PICOS framework, are reported in Supplementary Table 8 (Supplementary Section 2). Eligible studies were RCTs reporting on outcomes after at least 52 weeks of treatment as earlier outcomes were considered incomparable to the ELATIVE outcomes. Outcomes of interest were clinical efficacy, pruritus and safety outcomes, with the clinical efficacy outcomes including, but not limited to, survival, response rates or biochemical response. Pruritus outcomes included, but were not limited to, experience of pruritus, pruritus-specific health-related quality-of-life or ItchyQoL. Safety outcomes included, but were not limited to, adverse events, deaths or discontinuation due to adverse events.

The comparability of studies was assessed by identifying heterogeneity in study design, population, outcomes and treatments considered. Two clinical experts advised that age at diagnosis, ALP levels, TB, cirrhosis and antinuclear antibody positive status at baseline were treatment effect modifiers, and validated that they were comparable across the included studies for patients with PBC; the feasibility assessment was modified based on their insights.

### Risk-of-bias assessment

Risk-of-bias (RoB) in the studies included in the network meta-analysis (NMA) was assessed using Version-2 of the Cochrane RoB tool for randomized trials (RoB 2) [25].

### Statistical analysis

Despite both studies specifying ALP ≥ 1.67 × ULN and TB ≤ 2 × ULN as key eligibility criteria, the clinical heterogeneity assessment found that definitions of ULN for ALP and TB varied. The ALP ULN was 129 U/l and 104 U/l for males and females in ELATIVE, respectively, compared with 116 U/l in RESPONSE. The TB ULN was 1.2 mg/dl and 1.1 mg/dl in ELATIVE and RESPONSE, respectively. Individual patient data (IPD) was available for ELATIVE, whereas only published aggregate data was available for RESPONSE. To improve similarity of baseline characteristics across respective trial populations and end points, the ELATIVE population was truncated to align with the RESPONSE inclusion criteria:

- ALP ≥ 1.67 × ULN (defined as 1.67 × 116 U/l)
- TB ≤ 2 × ULN (defined as 2 × 1.1 mg/dl)

Prior to performing the ITC, all outcomes in ELATIVE were re-estimated in the truncated ELATIVE population (n = 145) applying the definitions of ULN for ALP and TB from RESPONSE to the outcome definitions. Further methodological details are reported in Section S3 of the Supplementary materials.

An anchored ITC was conducted via a Bayesian NMA using aggregate data from the truncated ELATIVE and RESPONSE populations in accordance with methodological framework for Pairwise and Network Meta-Analysis of Randomized Controlled Trials reported in the NICE Decision Support Unit (DSU) Technical Support Document 2 [26]. The network consisted of elafibranor, seladelpar and placebo, with placebo serving as the common comparator from the ELATIVE and RESPONSE studies. As clinical validation confirmed key treatment effect modifiers at baseline were sufficiently similar in the aggregated data, it was unnecessary to pursue techniques such as simulated or matching-adjusted treatment comparisons. All analyses were performed using outcomes in the intention-to-treat (ITT) populations after 52 weeks.

Aligning with ELATIVE and RESPONSE, the primary analysis was the likelihood of cholestasis response. An additional analysis of cholestasis response in patients with ALP ≥350 U/l at baseline was performed, aligning with the predefined subpopulation from RESPONSE. Further binary end points assessed included likelihood of ALP normalization, pruritus as a treatment-emergent adverse event (TEAE), and all-cause discontinuation. The continuous end points assessed included the mean change from baseline (CFB) in ALP and mean CFB in pruritus according to 5-D Itch, the PBC-40 itch domain and a numerical rating scale (NRS; pruritus NRS and worst-itch NRS for RESPONSE and ELATIVE, respectively). The latter three analyses were also performed in the pruritus ITT subpopulation (patients in the ITT population with a NRS ≥ 4 at baseline). For binary outcomes, odds ratios (ORs) were estimated, while for continuous outcomes, the difference in least-square mean in CFB were estimated; median outcomes with 95% credible intervals (CrIs) were reported in line with typical Bayesian methodology. Probabilities, derived from the full posterior distribution for each Bayesian model (referred to as ‘posterior probabilities’), were generated for all outcomes to quantify the likelihood that elafibranor treatment was more effective than placebo or seladelpar. To quantify statistical heterogeneity between the studies, the between-study standard deviation (SD) was calculated for random-effects models. Both random-effects (base case) and fixed-effect models (sensitivity analyses) were assessed. Informative priors were used for between-study SD and vague priors were used for treatment effects and baseline study effects.

OpenBUGS version 3.2.3 and R version 4.3.1 via RStudio were used to perform the NMA [27–29]. Additional methodological details are reported in Section S4 of the Supplementary materials.

## Results

### Systematic literature review

**Figure 1.**
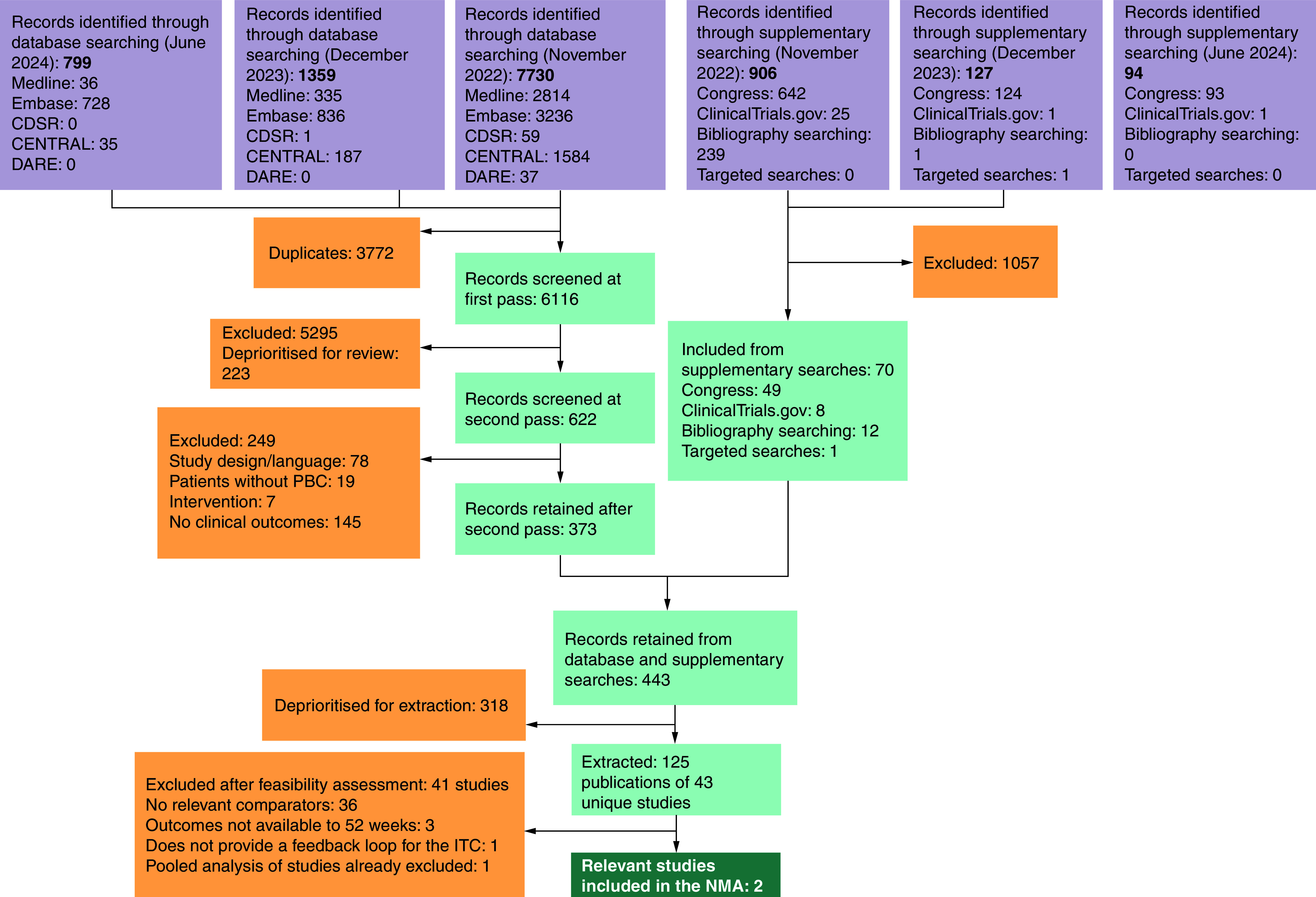
PRISMA diagram of studies identified in the systematic literature review. CDSR: Cochrane Database of Systematic Reviews; CENTRAL: Cochrane Central Register of Controlled Trials; DARE: Database of Abstracts of Reviews of Effects; NMA: Network meta-analysis; PBC: Primary biliary cholangitis.

Figure 1 presents the results of each stage of the screening process in the SLR. Searches of electronic databases retrieved 9,888 records, of which 3,772 were duplicates, resulting in 6,116 novel records that were screened at the title/abstract review stage.Following full-text screening, 125 publications reporting on 43 unique studies were included for extraction in the SLR following prioritization of RCTs in the second-line or later setting.

### Feasibility assessment

Of the 43 unique studies extracted, there were two elafibranor and five seladelpar studies. Five studies were excluded: three according to the NMA eligibility criteria, one could not provide a feedback loop in subsequent ITCs and one was a pooled analysis of already excluded studies (Supplementary Section 5). A summary of all studies excluded from the NMA is reported in Supplementary Table 9 (Supplementary Section 5).

**Figure 2.**
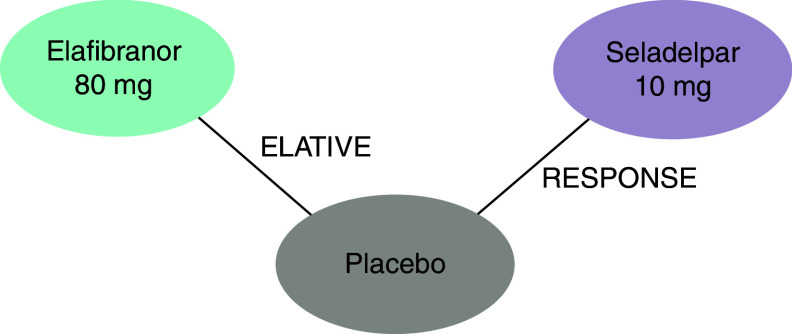
Network of evidence for the network meta-analysis. NMA: Network meta-analysis.

The network of evidence comprised ELATIVE (elafibranor; NCT04526665) and RESPONSE (seladelpar; NCT03301506) with the common comparator of placebo (Figure 2). Study design, population, outcomes and treatments were found to be sufficiently homogenous across both trials (Table 1). However, it was found that the inclusion and exclusion criteria of ELATIVE and RESPONSE were not aligned due to differences in the ULN definitions in ALP and TB. After the alignment of ULNs, the feasibility assessment concluded that comparisons between ELATIVE and RESPONSE were feasible and a NMA was appropriate.

**Table 1.** Summary of primary publications of the studies included in the network meta-analysis.

		ELATIVE (NCT04526665)		RESPONSE (NCT03301506)
**Summary of key study characteristics **				
Key eligibility criteria		Aged 18–75 Diagnosis of PBC with inadequate response to or unacceptable side effects with UDCA Taking UDCA for at least 12 months or unable to tolerate UDCA		
		ALP ≥ 1.67 × ULN (ULN = 104 U/l for females and 129 U/l for males) TB ≤2 × ULN (ULN = 1.2 mg/dl)		ALP ≥ 1.67 × ULN (ULN = 116 U/l) TB <2 × ULN (ULN = 1.1 mg/dl)
Interventions		Elafibranor 80 mg once daily (N = 108) Placebo once daily (N = 53)		Seladelpar 10 mg once daily (N = 128) Placebo once daily (N = 65)
Concomitant UDCA usage		95.0%		93.8%
Baseline characteristics, mean (SD)		ITT population		
		Before truncation	After truncation	
	Age at diagnosis (years)	49 (8.2)	48.4 (8.3)	49.2 (14.7)
	ALP (U/l)	322 (150.9)	338.0 (150.5)	314.3 (170.2)
	TB (mg/dl)	0.56 (0.3)	0.57 (0.3)	0.76 (0.4)
	Liver stiffness (kPa)	10.1 (8.2)	10.3 (8.3)	9.4 (7.5)
	Antinuclear antibody status	NR	NR	NR
		Pruritus ITT population		
	Age at diagnosis (years)	47.1 (8.3)	46.9 (8.4)	NR
	ALP (U/l)	335.1 (192.1)	357.1 (195.0)	NR
	TB (mg/dl)	0.61 (0.3)	0.63 (0.3)	NR
	Liver stiffness (kPa)	9.9 (6.8)	10.1 (6.5)	NR
	Antinuclear antibody status	NR	NR	NR
Outcomes analyzed		Cholestasis response ALP normalization Change in pruritus across various end points (NRS, 5-D Itch and PBC-40 Itch) Frequency of adverse events, including pruritus Patient disposition Change in non-invasive markers of fibrosis		
Study type		Phase III, double-blind, placebo-controlled, multicenter, randomized study		
**Risk-of-bias assessment: Cochrane's risk-of-bias tool for randomized trials 2.** ^**1**^				
1.Randomization				
1.1. Was the allocation sequence random?		Yes		Yes
1.2. Was the allocation sequence concealed until participants were enrolled and assigned to interventions?		Yes		Yes
1.3. Did baseline difference between intervention groups suggest a problem with the randomization process?		No		No
2. Deviations from intended interventions?				
2.1. Were participants aware of their assigned intervention during the trial?		No		No
2.2. Were carers and people delivering the interventions aware of participants' assigned intervention during the trial?		No		No
2.3. Were there deviations from the intended intervention that arose because of the trial context?^†^		N/A		N/A
2.4. Were these deviations likely to have affected the outcome?^†^		N/A		N/A
2.5. Were these deviations from intended deviation balanced between groups?^†^		N/A		N/A
2.6. Was an appropriate analysis used to estimate the effect of assignment to intervention?		Yes		Yes
2.7. Was there potential for a substantial impact (on the result) of the failure to analyze participants in the group to which they were randomized?^‡^		N/A		N/A
3. Missing outcome data				
3.1 Were data for this outcome available for all, or nearly all, participants randomized?		Yes		Yes
3.2. Is there evidence that the result was not biased by missing outcome data?^§^		N/A		N/A
3.3. Could missingness in the outcome depend on its true value?^§^		N/A		N/A
3.4. Is it likely that missingness in the outcome depended on its true value?^§^		N/A		N/A
4. Measurement of the outcome				
4.1. Was the method of measuring the outcome inappropriate?		No		No
4.2. Could measurement or ascertainment of the outcome have differed between intervention groups?		No		No
4.3. Were outcome assessors aware of the intervention received by study participants?		No		No
4.4. Could assessment of the outcome have been influenced by knowledge of intervention received?^¶^		N/A		N/A
4.5. Is it likely that assessment of the outcome was influenced by knowledge of intervention received?^¶^		N/A		N/A
5. Selection of the reported result				
5.1. Were the data that produced this result analyzed in accordance with a prespecified analysis plan that was finalized before unblinded outcome data were available for analysis?		Yes		Yes
5.2. Is the numerical result being assessed likely to have been selected, on the basis of the results, from multiple eligible outcome measurements (e.g., scales, definitions, time points) within the outcome domain?		No		No
5.3 Is the numerical result being assessed likely to have been selected, on the basis of the results, from multiple eligible analyses of the data?		No		No
Overall risk of bias		Low		Low

The subdomains of the RoB tool do not need completing based on the following conditions.

† Where the response to 2.2 is no.

‡ Where the response to 2.6 is yes.

§ Where the response to 3.1 is no.

¶ Where the response to 4.3 is no.

ALP: Alkaline phosphatase; ITT: Intention-to-treat; N/A: Not applicable; NRS: Numerical rating scale; PBC: Primary biliary cholangitis; TB: Total bilirubin; UDCA: Ursodeoxycholic acid; ULN: Upper limit of normal.

Data taken from [25].

### RoB assessment

The assessment found a low RoB across the two trials (Table 1).

### Network meta-analysis results

Following IPD analysis of ELATIVE to harmonize the ELATIVE and RESPONSE patient populations in terms of trial eligibility criteria, 16 patients were excluded as their baseline ALP did not meet the RESPONSE eligibility criteria. The results of the IPD analysis of ELATIVE and the data used in the ITC analyses are reported in Sections S6 & S7 of the Supplementary materials, respectively.

**Figure 3.**
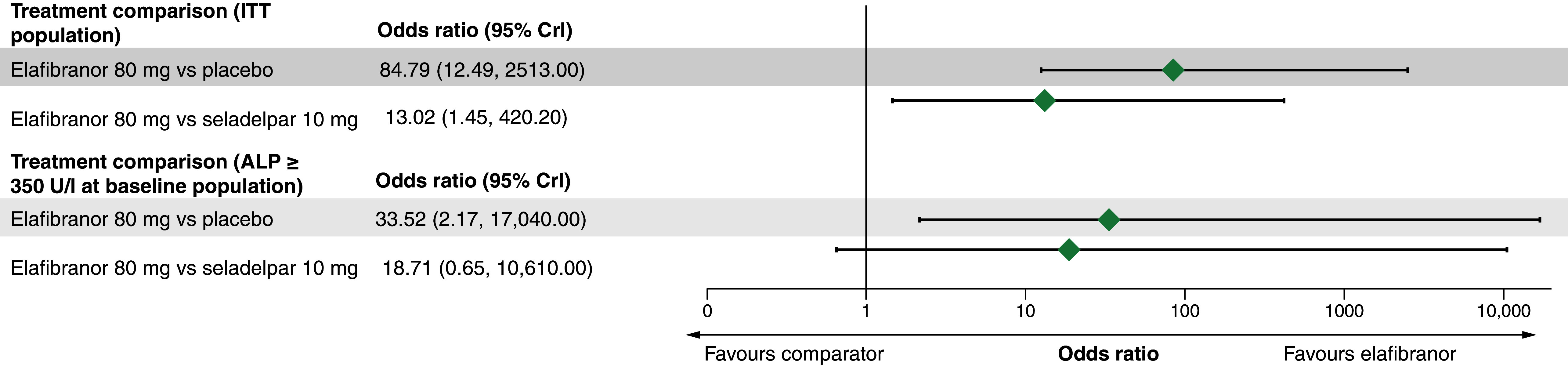
Odds ratio of achieving cholestasis response at 52 weeks (random-effects model). ALP: Alkaline phosphatase; CFB: Change from baseline; CrI: Credible interval; ITT: Intention-to-treat; mg: Milligram; U/l: Units per liter.

Using placebo as the common comparator in the random-effects NMA, there were greater odds of achieving cholestasis response at 52 weeks in the ITT population for elafibranor-treated patients than placebo- (median OR [95% CrI]: 84.79 [12.49, 2513.00]) or seladelpar-treated patients (13.02 [1.45, 420.20]; Figure 3). The posterior probabilities that the odds were higher with elafibranor than placebo or seladelpar were 100.0% and 99.1%, respectively (Table 2).

**Table 2.** Summary statistics from the base-case network meta-analysis^†^.

Analysis	Between-study SD on mean difference or OR scale^‡^	Total residual deviance	Posterior probability of elafibranor being preferred to placebo	Posterior probability of elafibranor being preferred to seladelpar
Cholestasis response (ITT population)	0.301	3.495	1.000	0.991
Cholestasis response (ALP ≥ 350 U/l at baseline population)	0.312	4.371	0.997	0.952
ALP normalization	0.301	3.851	0.999	0.380
Change from baseline in ALP	11.810	3.333	1.000	0.472
Pruritus as a TEAE	0.305	4.062	0.816	0.649
All-cause discontinuation	0.194	3.430	0.586	0.368
Change from baseline in a pruritus NRS (ITT)	0.205	3.365	0.850	0.236
Change from baseline in a pruritus NRS (Pruritus ITT)	0.248	3.356	0.950	0.333
Change from baseline in 5-D Itch (ITT)	0.366	3.344	0.969	0.233
Change from baseline in 5-D Itch (Pruritus ITT)	0.491	4.013	0.995	0.448
Change from baseline in PBC-40 Itch (ITT)	0.272	3.347	0.980	0.642
Change from baseline in PBC-40 Itch (Pruritus ITT)	0.313	3.358	0.992	0.640

† Between-study SD on mean difference scale was used for continuous outcomes, while the OR scale was used for binary outcomes.

‡ The base-case model used a random-effects model.

ALP: Alkaline phosphatase; CFB: Change from baseline; ITT: Intention-to-treat; LSM: Least-square mean; NMA: Network meta-analysis; NRS: Numerical rating scale; OR: Odds ratio; PBC: Primary biliary cholangitis; SD: Standard deviation; TEAE: Treatment-emergent adverse event; U/l: Units per liter.

Consistent with the ITT population, there were greater odds of achieving cholestasis response at 52 weeks in the ALP ≥ 350 U/l subpopulation for elafibranor-treated patients than placebo- (33.52 [2.17, 17,040.00]) or seladelpar-treated patients (18.71 [0.65, 10,610.00]; Figure 3). The posterior probabilities that the odds were higher with elafibranor than placebo or seladelpar were 99.7% and 95.2%, respectively (Table 2).

**Figure 4.**
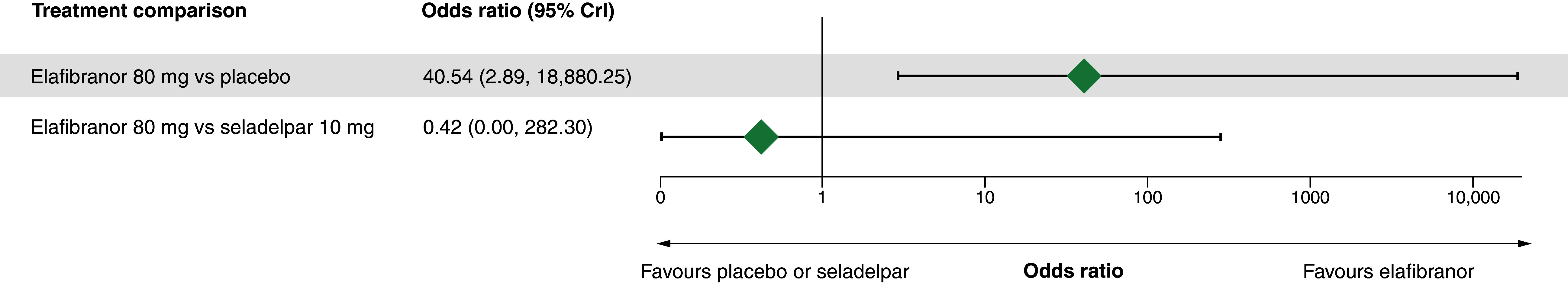
Odds ratio of achieving alkaline phosphatase normalization at 52 weeks in the intention-to-treat population (random-effects model). ALP: Alkaline phosphatase; CrI: Credible interval; ITT: Intention-to-treat; mg: Milligram.

**Figure 5.**
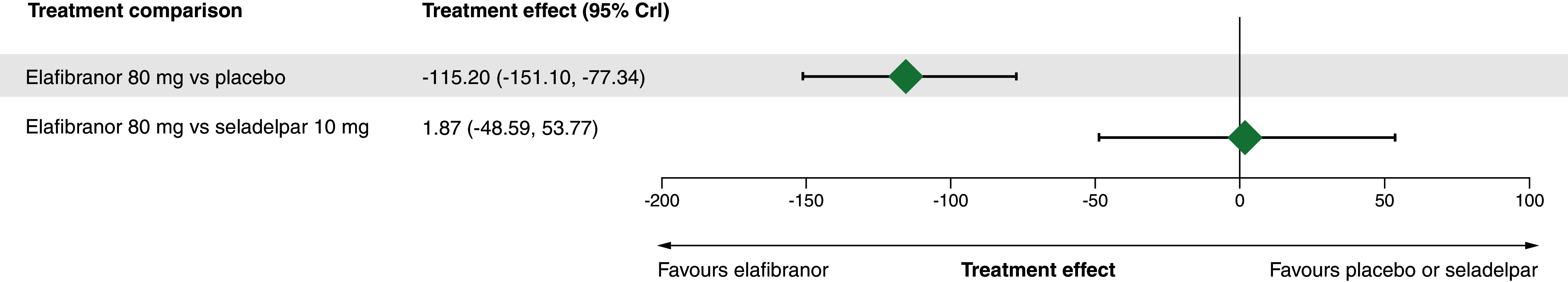
Median difference in least-square mean change from baseline in alkaline phosphatase at 52 weeks in the intention-to-treat population (random-effects model). ALP: Alkaline phosphatase; CFB: Change from baseline; CrI: Credible interval; ITT: Intention-to-treat; LSM: Least-square mean; mg: Milligram.

The median OR (95% CrI) of ALP normalization at 52 weeks for elafibranor-treated patients compared with placebo- or seladelpar-treated patients were 40.54 (2.89, 18,880.25) and 0.42 (0.00, 282.30), respectively (Figure 4). There were 99.9% and 33.8% posterior probabilities that the odds were higher with elafibranor than placebo or seladelpar, respectively (Table 2). Likewise, the median difference in least-square mean CFB (95% CrI) in ALP at 52 weeks for elafibranor-treated patients compared with placebo- or seladelpar-treated patients were -115.20 (-151.10, -77.34) and 1.87 (-48.59, 53.77), respectively (Figure 5). There were 100.0% and 47.2% posterior probabilities that the reduction was greater for elafibranor than placebo or seladelpar, respectively (Table 2).

**Figure 6.**
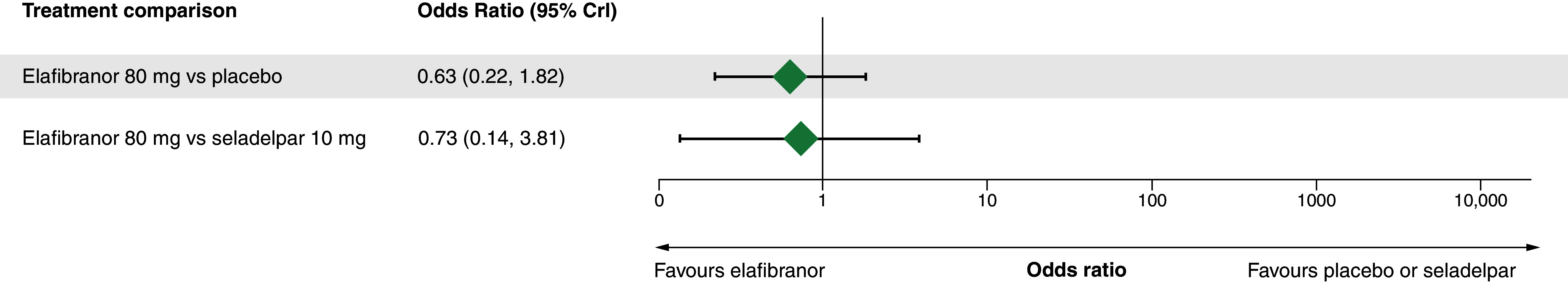
Odds ratio of occurrence of pruritus as a treatment-emergent adverse event at 52 weeks in the intention-to-treat population (random-effects model). CrI: Credible interval; ITT: Intention-to-treat; mg: Milligram; TEAE: Treatment-emergent adverse event.

**Figure 7.**
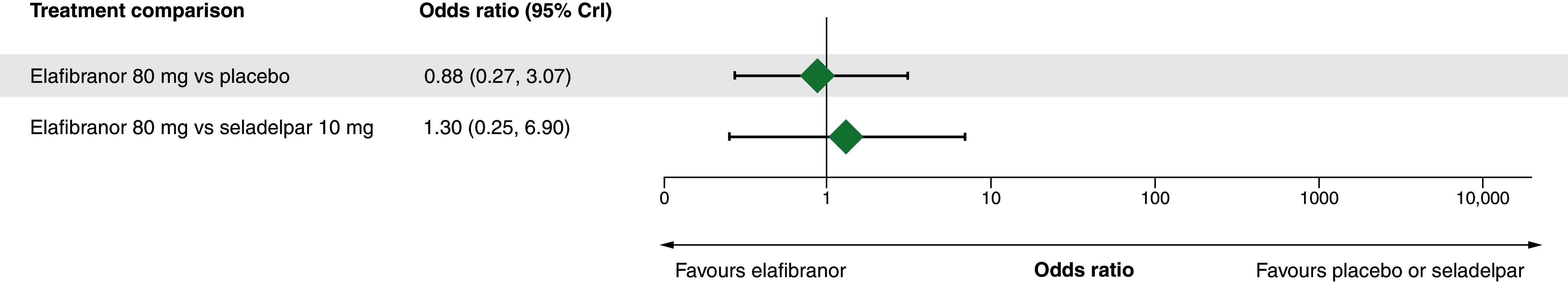
Odds ratio of all-cause discontinuation at 52 weeks in the intention-to-treat population (random-effects model). CrI: Credible interval; ITT: Intention-to-treat; mg: Milligram.

**Figure 8.**
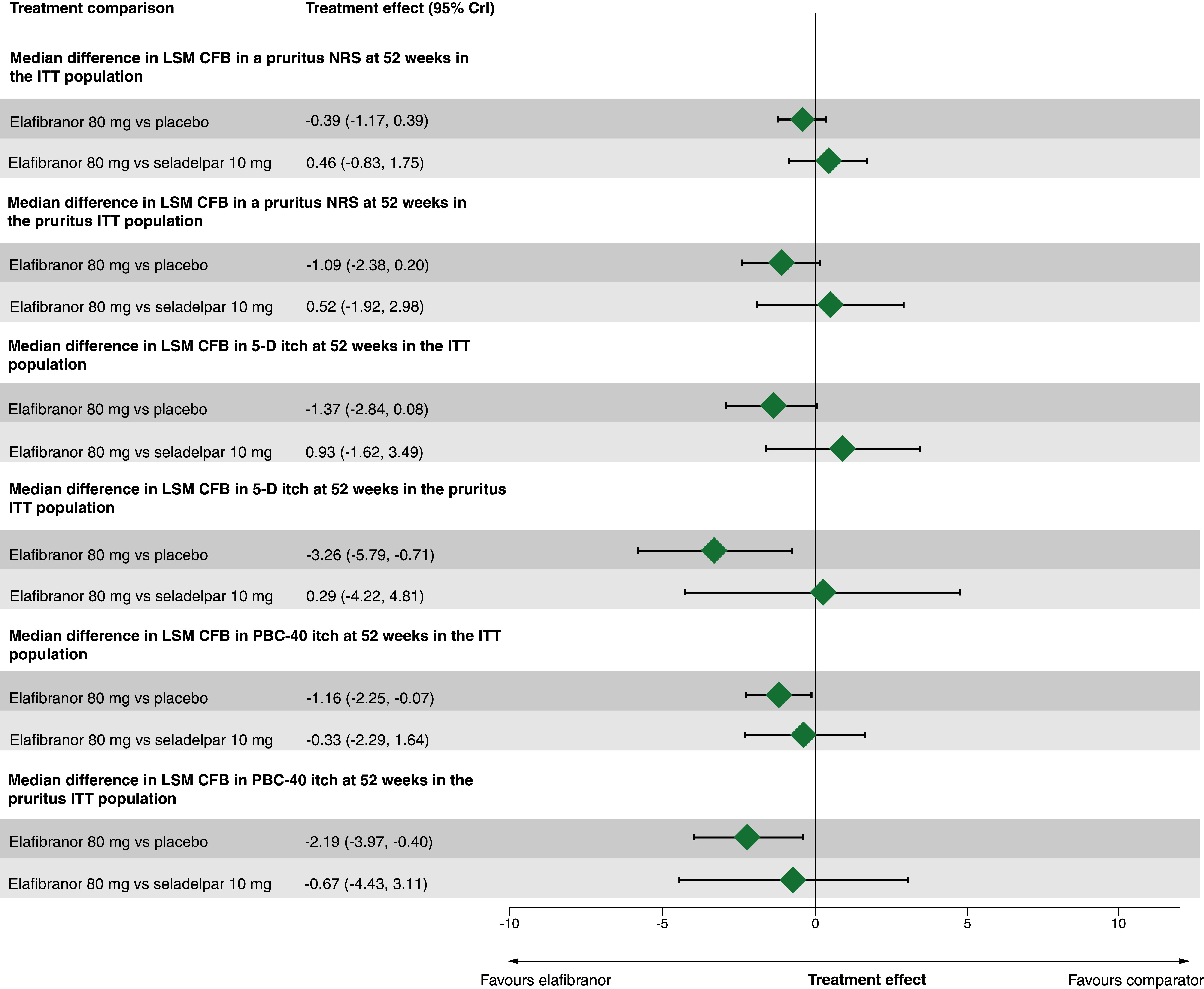
Median difference in least-square mean change from baseline for pruritus outcomes at 52 weeks (random-effects models). ALP: Alkaline phosphatase; CFB: Change from baseline; CrI: Credible interval; ITT: Intention-to-treat; LSM: Least-square mean; mg: Milligram; NRS: Numerical rating scale; PBC: Primary biliary cholangitis.

The median relative treatment effects and 95% CrIs for pruritus as a TEAE, all-cause discontinuation or CFB in pruritus when measured using a NRS, 5-D Itch or the itch domain of the PBC-40 questionnaire at 52 weeks indicated substantial uncertainty in elafibranor’s relative effectiveness versus seladelpar (Figures 6–8). The posterior probabilities that elafibranor-treated patients had preferred outcomes to seladelpar-treated patients in these outcomes ranged from 23.6 to 64.9% (Table 2), supporting the uncertainty in relative treatment effects. Relative effect matrices are reported in Section S9 of the Supplementary materials.

The models were a good fit to the data, as demonstrated by the total residual deviance remaining below the number of data points in all but three analyses; total residual deviance was marginally greater than the number of data points in the analyses of pruritus as a TEAE, CFB in pruritus measured using 5-D Itch in the pruritus ITT subgroup and cholestasis response in the ALP ≥350 U/l subpopulation (Table 2 & Supplementary Section 8). Using between-study SD to assess heterogeneity, high levels of heterogeneity were not identified, while moderate heterogeneity was identified for cholestasis response and ALP normalization. There were low levels of heterogeneity identified for the remaining binary outcomes. Low levels of heterogeneity were observed for the continuous outcomes when assessing the between-study SD on the standardized mean difference scale. The burn-in, thinning interval and number of iterations for each analysis are reported in Supplementary Table 16 (Supplementary Section 10).

Results from the fixed-effect models (Supplementary Section S11) were consistent with results from the base case random-effects models. As expected, the fixed-effect models had narrower CrIs for the relative treatment effects. The random-effects models were retained as the base-case because the difference in total residual deviance did not suggest a better fit to the data with the fixed-effect model.

## Discussion

Despite wide CrIs suggesting uncertainty in the magnitude of the relative effects of elafibranor compared with seladelpar, there were 99.1% and 95.2% probabilities that elafibranor was more effective in achieving cholestasis response at week 52 in the ITT and ALP ≥ 350 U/l at baseline populations, respectively. This provides strong evidence for elafibranor treatment benefit compared with seladelpar for this outcome. For all other outcomes including ALP normalization, reduction in ALP from baseline, all-cause discontinuation, reduction in pruritus from baseline in pruritus, and pruritus as an adverse event, probabilities that outcomes were more favorable with elafibranor ranged between 23.6% and 64.9%, providing no strong evidence of different treatment effects between elafibranor and seladelpar.

Trial comparability was assessed by examining heterogeneity in study design, population, outcomes and treatments administered. Both ELATIVE and RESPONSE had high-quality trial design, with double-blind, placebo-controlled, multicenter, multinational trials assessed as low RoB. Although ULN definitions for ALP and TB differed, access to IPD allowed exclusion of ELATIVE patients not meeting the stricter RESPONSE eligibility criteria and re-estimation of outcomes using RESPONSE thresholds. This approach taken avoided the limitations of simulated or matching-adjusted treatment comparisons where differences in outcome definitions cannot be accounted for and facilitated a robust NMA using aggregated data without need for an IPD meta-analysis.

In aligning to RESPONSE, patients with the lowest baseline ALP were excluded from ELATIVE. Therefore, the cohort considered was less likely to achieve cholestasis response than the ELATIVE ITT cohort, as demonstrated by a monotonically decreasing likelihood of cholestasis response with increasing baseline ALP (≤2, 2–≤2.5, 2.5–≤3, 3–≤4 and >4 × ULN) for elafibranor- or placebo-treated patients in subgroup analyses of ELATIVE [30]. It is also not reported which central laboratory or which assays were used in RESPONSE to determine biological markers. Therefore, it is unclear whether there are differences leading to the estimation of response in both studies. These differences underscore the challenges in comparing outcomes across trials with varying inclusion criteria and response thresholds. Indeed, between-study SD demonstrated moderate heterogeneity for cholestasis response and ALP normalization, suggesting potential underlying differences which cannot be accounted for.

For TB, no patients were excluded from ELATIVE for not meeting the RESPONSE TB eligibility criteria. Given this, mean baseline TB remained lower in ELATIVE compared with RESPONSE. However, as the mean TB in both trials was below the ULN, no additional adjustment was performed. This decision was guided by clinical input and supported by major prognostic models, which demonstrate risk stratification primarily when TB exceeds the ULN [10].

The feasibility assessment examined outcome definitions in ELATIVE and RESPONSE as part of the heterogeneity assessment and found that outcomes were similarly defined, supporting the validity of comparing these end points across trials. In addition, because ALP and TB were identified as treatment effect modifiers their similarity at baseline was confirmed. This reduces the risk of bias when comparing response-based end points, as patients had comparable potential for improvement on these biomarkers. Conversely, pruritus, which was assessed using the patient reported outcomes of a NRS, 5-D Itch and the itch domain of the PBC-40 questionnaire, was not identified as a treatment effect modifier. As a result, differences between studies in baseline levels could influence the achievable amount of improvement that may be observed. Despite this, there was a comparable proportion of patients with a NRS ≥4 at baseline in the two trials (41% in ELATIVE compared with 36% in RESPONSE). However, as a subjective outcome, pruritus may be associated with more between-study variation than response-based end points. Though informed priors for between-study SD were used to mitigate this, limited study data restricted the model’s ability to fully account for potential between-study differences and findings should be interpreted with caution. However, by prespecifying the analyses for this NMA in a statistical analysis plan and focusing on aligning end point definitions and analytic approaches aligned with those of the original trials, the risk of confounding in the analyses was minimized.

Considering the evidence informing the NMA, a small network led to uncertain relative treatment effects. Notably, recruiting a large study population of patients with advanced PBC was difficult, a subgroup of an already small patient population [31]. Therefore, though the studies were adequately powered to detect differences in their key end points versus placebo, the ability to achieve statistical power for ITCs may be limited [10,32]. Furthermore, excluding patients from ELATIVE not meeting the eligibility criteria of RESPONSE may have restricted the power of the analyses.

In addition to small numbers of patients contributing to the network of evidence, the network contained only one study per treatment comparison. This made it infeasible to estimate between-study SD. However, due to the limited data available for the analysis, random effects models with informed priors for between-study SD were used in the base case analyses throughout, unless there was evidence favoring the fixed effect analysis. Though this avoided overstating the precision of the results, it is a limitation of the analyses.

The lack of long-term clinical trials was also noted. To address the small number of studies included in the analysis, extra studies could have been included if analyses were conducted comparing outcomes after 12 weeks of treatment. However, given the progressive nature of PBC, longer follow-up was required to demonstrate the impact of treatment and these were prioritized for inclusion. Ongoing studies may provide further data in the future (Supplementary Table 19). Additionally, per-protocol population analyses were not possible due to insufficient data availability from RESPONSE.

The ITC results should be considered within the context of these limitations and the potential for unobserved biases. Moreover, for the binary outcomes, despite ORs being the natural output of an NMA, caution is warranted to avoid interpreting the ORs as risk ratios. Odds ratios, when used as a proxy, may overestimate risk ratios, though a ratio of 1 indicates no evidence of relative difference between treatments. Despite these limitations, the credibility of the NMA findings is supported by the similarity in key eligibility criteria, concomitant UDCA usage, baseline characteristics and outcome specifications across studies (Table 1). Further, the NMA results are validated by the consistency of the results with findings from ELATIVE and RESPONSE.

A recently published frequentist NMA by Giannini *et al.* [33] also evaluated the relative efficacy of elafibranor and seladelpar via a frequentist NMA with cholestasis response as the primary outcome, confirming that cholestasis response was statistically significantly more likely in elafibranor-treated patients than seladelpar-treated patients. Like this analysis, it also showed no significant differences in the occurrence of pruritus between elafibranor and seladelpar. A methodological advantage that this analysis has over that reported by Giannini *et al.* is that our Bayesian model provides full posterior distributions for treatment effects. While frequentist analyses produce p-values, which can be misinterpreted, Bayesian analyses enable probabilistic statements for direct interpretation of the probability that outcomes are better with one treatment compared with another. Bayesian models are also particularly advantageous in sparse networks, where frequentist methods can suffer from unstable variance estimation. Moreover, use of IPD allowed end point and trial eligibility harmonization for differences in ULN threshold definitions between studies. This harmonization is not possible in aggregate-data NMAs, and as such this analysis addresses a key limitation of Giannini *et al.*‘s by leveraging IPD from ELATIVE to align definitions for the ULN of ALP and TB to make ELATIVE and RESPONSE data more comparable.

Another NMA comparing treatments for patients with PBC refractory to UDCA has been published by Lin *et al.* which, like this analysis, found elafibranor to be associated with better biochemical response outcomes than seladelpar [34]. However, as the NMA performed by Lin *et al.* was informed by a SLR conducted in 2023, the RESPONSE study was not included. Therefore, the data informing comparisons of elafibranor and seladelpar differs across this analysis and that published by Lin *et al.*, Additionally, there are significant methodological differences between the two analyses, which limit the ability to reliably compare the conclusions of the two ITCs.

In conclusion, this Bayesian NMA provided strong probabilistic evidence that elafibranor-treated patients were more likely to achieve cholestasis response than seladelpar-treated patients. There was no strong evidence of different treatment effects between elafibranor and seladelpar for all other outcomes. These findings suggest that elafibranor is a promising second-line treatment option for adults with PBC, helping to alleviate disease burden. To strengthen the findings from this study, further analyses could be explored including additional end points and subgroup analyses to further evaluate the comparative effectiveness of elafibranor and seladelpar. Moreover, direct comparator studies are needed to validate the analysis’ findings.

## Summary points

- Summary of established knowledge on this subject:
  ○ Uncontrolled, primary biliary cholangitis (PBC) may progress with accumulation of symptoms such as pruritus (itching) and fatigue with significant impact on health-related quality-of-life. As PBC advances, the risk of complications necessitating liver transplant rise, such as hepatocellular carcinoma, persistent jaundice, hepatic decompensation and pruritus refractory to medical therapy.
  ○ Up to 40% of patients with PBC do not adequately respond to ursodeoxycholic acid (UDCA), the only recommended first-line treatment, and a further 3–5% of patients are UDCA-intolerant, highlighting a need for effective second-line treatments.
  ○ Elafibranor and seladelpar – both peroxisome proliferator-activated receptor agonists – have recently been approved by the US FDA, EMA and Medicine and Healthcare products Regulatory Agency to treat patients with PBC who have inadequately responded to, or are unable to tolerate, UDCA.
  ○ At present, the lack of head-to-head trials of elafibranor and seladelpar poses challenges for decision-makers in evaluating their relative efficacy and safety. Given this, an indirect treatment comparison (ITC) can be useful for synthesizing evidence to inform decision-making.
- What are the significant and/or new findings of this study?
  ○ The ELATIVE and RESPONSE randomized controlled trials were included in a novel, high-quality ITC to compare the relative efficacy and safety of elafibranor and seladelpar. Before undertaking the ITC with aggregated data, individual patient data from ELATIVE were used to align definitions for upper limit of normal of alkaline phosphatase (ALP) and total bilirubin across studies to make them more comparable. Results from the ITC analyses were consistent with existing evidence.
  ○ Elafibranor-treated patients had greater odds of achieving cholestasis response at 52 weeks than seladelpar-treated patients, with ≥99% posterior probability that the odds were higher for elafibranor than seladelpar, providing strong evidence supporting elafibranor’s treatment benefit. Results for this outcome were consistent in the subgroup of patients with baseline ALP ≥ 350 U/l.
  ○ The credible intervals around median treatment effects for all other outcomes after 52 weeks of treatment, including ALP normalization, mean change from baseline in ALP, all-cause discontinuation, mean change from baseline in pruritus, and pruritus as an adverse event, demonstrated considerable uncertainty in the relative effects of elafibranor versus seladelpar and provided no strong evidence of a difference between treatments.

## Supplementary Material


