# Supplementary material for: Network meta-analysis: relative clinical efficacy and safety of elafibranor versus seladelpar as second-line treatment for patients with primary biliary cholangitis
Source: J Comp Eff Res. 2026 Apr 17;15(5):e250206. doi: 10.57264/cer-2025-0206 (PMC13154925; doi:10.57264/cer-2025-0206)
Supplement: Supplementary file 1 [file cer-15-250206-s1.docx]

Table of Contents

[Table of Contents 1](#_Toc224039563)

[S1. SLR methods 2](#_Toc224039564)

[S1.S1. SLR eligibility criteria 2](#_Toc224039565)

[S1.S2. SLR search strategy 4](#_Toc224039566)

[S2. NMA eligibility criteria 12](#_Toc224039567)

[S3. Method to align ELATIVE trial data to RESPONSE 13](#_Toc224039568)

[S4. NMA analysis methodology 14](#_Toc224039569)

[S5. Studies excluded from the NMA 16](#_Toc224039570)

[S6. Summary of IPD analysis of ELATIVE 27](#_Toc224039571)

[S7. Summary of data included in the NMA 29](#_Toc224039572)

[S8. Further random-effects model results 30](#_Toc224039573)

[S9. Treatment effect matrices 32](#_Toc224039574)

[S10. Random-effects model settings 35](#_Toc224039575)

[S11. Fixed-effect model results 36](#_Toc224039576)

[S12. Ongoing studies of elafibranor and seladelpar 42](#_Toc224039577)

[S13. PRISMA checklist 43](#_Toc224039578)

[S14. References 47](#_Toc224039579)

## SLR methods

The eligibility criteria for the systematic literature review (SLR) which was used to inform the network meta-analysis (NMA) is reported in Table S 1.

The SLR was developed using literature searches up to 19^th^ June 2024. The SLR was informed by two earlier versions, with database search strategies reported in Table S 2 and Table S 3; searches were initially conducted on 21^st^ November 2022 (original SLR) and subsequently updated on 18^th^ December 2023 (SLR Update I) and 19^th^ June 2024 (SLR Update II). The study design terms were adapted from search filters published by the Scottish Intercollegiate Guidelines Network. The conference proceedings search strategies are reported in Table S 4 to Table S 6; searches were initially conducted on 13^th^ December 2022 (original SLR), 2^nd^ February 2024 (SLR Update I) and 22^nd^ July 2024 (SLR Update II). The search strategy for clinical trial registry searches are reported in Table S 7; searches were initially conducted on 19^th^ December 2022 (original SLR), 5^th^ December 2023 (SLR update I) and 12^th^ July 2024 (SLR update II).

### SLR eligibility criteria

**Table S 1****: Eligibility criteria for included publication in SLR**

| **Study characteristics** | **Inclusion** | **Exclusion** |
| --- | --- | --- |
| **P**opulation | - Unselected adult patients (aged ≥18 years) with PBC | - Patients without PBC - Children or adolescents (<18 years old) |
| **I**ntervention/ **C**omparator | - Monotherapy or combination therapy in any treatment line including, but not limited to: - Elafibranor - UDCA - OCA - Fibrates - Other future competitors for elafibranor e.g., seladelpar | - No planned intervention |
| **O**utcomes | - Clinical efficacy/effectiveness outcomes, including but not limited to: - Survival outcomes - Response rates - Biochemical responses (e.g., CFB in ALP levels, etc.) - Patients experiencing pruritus - Safety outcomes, including but not limited to: - Adverse events (AEs) (including TRAEs and serious AEs) - Deaths - Discontinuation due to AEs/TRAEs - Pruritus-specific health-related QoL outcome measures such as: - ItchyQoL | - Studies not reporting any relevant outcomes, e.g., pharmacokinetics. - Studies reporting a relevant outcome but in a mixed population where results for PBC are not reported separately. |
| **S**tudy Design | - RCTs of any intervention - Non-randomised interventional studies featuring elafibranor - Non-randomised interventional studies (e.g., single-arm trials) featuring any other interventions within scope - Observational studies   ***Due to the high volume of identified evidence, a two-stage prioritisation strategy was ultimately implemented:***   1. *Interventional and observational studies in the second-line or later treatment setting were prioritised following the title/abstract review stage (all other observational studies were deprioritised and not reviewed at the full-text stage)* 2. *Any studies of elafibranor, and RCTs in the second-line or later treatment setting were then prioritised for extraction* | - Non-primary research publications, including: - Narrative reviews - Editorials - Guidelines - Commentaries - Opinion pieces |
|  | Relevant SLRs and (N)MAs would be considered at the title/abstract review stage and hand searched for relevant primary studies, but will be excluded during the full-text review stage unless they report primary research | |
| Language | - English | - Non-English |
| Geography | - Global | - None |
| Publication types & dates | - Peer-reviewed literature or congress abstracts published in or after 2021 - Studies on human subjects - Studies with an abstract or full-text published in the English language | - Peer-reviewed literature or congress abstracts published prior to 2021 - In vitro/animal studies |

Abbreviations: AE – adverse event; ALP – alkaline phosphatase; NMA – network meta-analysis; PBC – Primary Biliary Cholangitis; QoL – quality of life; RCT – randomised control trial; SLR – systematic literature review; TRAE – treatment related adverse event

### SLR search strategy

**Table S 2****: Search terms used for MEDLINE (searched via the Ovid SP platform for the original SLR and SLR Update I)**

| **Term Group** | **#** | **Searches** | **Results 21.11.2022** | **Results 18.12.2023** |
| --- | --- | --- | --- | --- |
| Disease area: primary biliary cholangitis |  | *Liver Cirrhosis, Biliary/ | 6690 | 6895 |
|  |  | (primary biliary cholangitis or primary biliary cholestasis or primary biliary cirrhosis or PBC).ti,ab,kf. | 11323 | 11882 |
|  |  | 1 or 2 | 12480 | 13050 |
| Interventional studies (RCTs and non-RCTs) |  | Randomized Controlled Trials as Topic/ | 158804 | 165474 |
|  |  | Randomized Controlled Trial/ | 581137 | 604215 |
|  |  | Random Allocation/ | 106894 | 107041 |
|  |  | Double-Blind Method/ | 173639 | 176795 |
|  |  | Single-Blind Method/ | 32303 | 33081 |
|  |  | Placebos/ | 35924 | 35935 |
|  |  | exp Clinical Trials as topic/ | 378425 | 386313 |
|  |  | Clinical Trial/ | 536481 | 539133 |
|  |  | Clinical Trial, Phase I/ or Clinical Trial, Phase II/ or Clinical Trial, Phase III/ or Clinical Trial, Phase IV/ | 78653 | 82298 |
|  |  | Controlled Clinical Trial/ or Adaptive Clinical Trial/ | 95139 | 95510 |
|  |  | randomized controlled trial.pt. | 581137 | 604215 |
|  |  | clinical trial.pt. | 536481 | 539133 |
|  |  | (clinical trial, phase i or clinical trial, phase ii or clinical trial, phase iii or clinical trial, phase iv).pt. | 78653 | 82298 |
|  |  | (controlled clinical trial or multicenter study).pt. | 419005 | 432168 |
|  |  | (clinical adj trial$).ti,ab,kf. | 466974 | 508526 |
|  |  | ((singl$ or doubl$ or treb$ or tripl$) adj (blind$3 or mask$3)).ti,ab,kf. | 192862 | 201304 |
|  |  | placebo$.ti,ab,kf. | 241949 | 252567 |
|  |  | (allocat$ adj2 random$).ti,ab,kf. | 42013 | 45098 |
|  |  | Randomi?ed adj2 trial$.ti,ab,kf. | 407080 | 446042 |
|  |  | rct.ti,ab,kf. | 31749 | 35777 |
|  |  | (single arm adj3 (trial$ or stud$)).ti,ab,kf. | 8388 | 9945 |
|  |  | (open label adj (trial$ or stud$)).ti,ab,kf. | 13048 | 13768 |
|  |  | (non blinded adj (trial$ or stud$)).ti,ab,kf. | 224 | 243 |
|  |  | (pragmatic trial$ or pragmatic stud$).ti,ab,kf. or Pragmatic Clinical Trial/ | 4246 | 4664 |
|  |  | or/4-27 | 1983660 | 2079478 |
| Observational studies |  | Epidemiologic Studies/ | 9193 | 9443 |
|  |  | Observational Study/ | 134780 | 149384 |
|  |  | Cohort Studies/ | 321739 | 335173 |
|  |  | exp Case-Control Studies/ | 1369979 | 1462987 |
|  |  | Cross-Sectional Studies/ | 446870 | 485289 |
|  |  | Clinical Study/ | 5331 | 5716 |
|  |  | Follow-Up Studies/ | 688450 | 694423 |
|  |  | Longitudinal Studies/ | 161468 | 168223 |
|  |  | Retrospective Studies/ | 1074257 | 1162360 |
|  |  | Prospective Studies/ not Randomized Controlled Trials as Topic/ | 636789 | 666225 |
|  |  | (observational adj (study or studies)).ti,ab,kf. | 150834 | 171221 |
|  |  | (cohort adj (study or studies)).ti,ab,kf. | 296874 | 338391 |
|  |  | cohort analy$.ti,ab,kf. | 11797 | 13284 |
|  |  | case control.ti,ab,kf. | 149419 | 159415 |
|  |  | cross sectional.ti,ab,kf. | 479274 | 538561 |
|  |  | (follow up adj (study or studies)).ti,ab,kf. | 56705 | 59248 |
|  |  | longitudinal.ti,ab,kf. | 310639 | 339180 |
|  |  | retrospective.ti,ab,kf. | 700115 | 778500 |
|  |  | (chart adj3 review$).ti,ab,kf. | 52057 | 55944 |
|  |  | exp Registries/ | 115210 | 118720 |
|  |  | (registry or registries).ti,ab,kf. | 167044 | 184293 |
|  |  | (prospective adj (study or studies)).ti,ab,kf. | 202477 | 214167 |
|  |  | (epidemiologic$ adj (study or studies)).ti,ab,kf. | 93730 | 97592 |
|  |  | (evaluation adj (study or studies)).ti,ab,kf. | 7144 | 7723 |
|  |  | (chart adj3 review$).ti,ab,kf. | 52057 | 55944 |
|  |  | or/29-53 | 3900849 | 4164577 |
| Exclusion terms |  | exp animals/ not exp humans/ | 5067266 | 5175456 |
|  |  | (comment or editorial or case reports or historical article).pt. | 4052655 | 4197494 |
|  |  | (case stud$ or case report$).ti. | 362167 | 395457 |
|  |  | or/55-57 | 9111076 | 9368540 |
| Combined |  | 3 and (28 or 54) | 2969 | 3144 |
|  |  | 59 not 58 | 2814 | 2981 |
| **Date limit** |  | limit 60 to yr=2022-current | **-** | 328 |

Databases: Ovid MEDLINE(R) and Epub Ahead of Print, In-Process, In-Data-Review & Other Non-Indexed Citations and Daily 1946 to November 21st 2022 (original SLR) and 1946 to December 4th 2023 (updated SLR).

Abbreviations: PBC, primary biliary cholangitis; RCT, randomised controlled trial.

**Table S 3****: Search terms used in MEDLINE, Embase, CDSR and CENTRAL (via Ovid SP) in SLR Update II**

| **Term Group** | **#** | **Searches** | **Results 19.06.2024** |
| --- | --- | --- | --- |
| Disease area: primary biliary cholangitis | 1 | *Liver Cirrhosis, Biliary/ or *biliary liver cirrhosis/ or "liver cirrhosis, biliary".kw. | 9462 |
|  | 2 | (primary biliary cholangitis or primary biliary cholestasis or primary biliary cirrhosis or PBC).ti,ab,kf,kw. | 32139 |
|  | 3 | 1 or 2 | 34060 |
| Interventional studies (RCTs and non-RCTs) | 4 | Randomized Controlled Trials as Topic/ or "randomized controlled trial (topic)"/ | 492407 |
|  | 5 | Randomized Controlled Trial/ | 1443716 |
|  | 6 | Random Allocation/ or randomization/ | 232820 |
|  | 7 | Double-Blind Method/ or double blind procedure/ | 570342 |
|  | 8 | Single-Blind Method/ or single blind procedure/ | 116158 |
|  | 9 | crossover procedure/ | 78461 |
|  | 10 | Placebos.mp. or placebo/ [mp=ti, bt, ab, ot, nm, hw, fx, kf, ox, px, rx, ui, sy, ux, mx, tn, dm, mf, dv, dq] | 483508 |
|  | 11 | exp Clinical Trials as Topic/ or exp "clinical trial (topic)"/ | 949383 |
|  | 12 | Clinical Trial/ | 1624297 |
|  | 13 | Clinical Trial, Phase I/ or phase 1 clinical trial/ or Clinical Trial, Phase II/ or phase 2 clinical trial/ or Clinical Trial, Phase III/ or phase 3 clinical trial/ or Clinical Trial, Phase IV/ or phase 4 clinical trial/ | 329011 |
|  | 14 | Controlled Clinical Trial/ or Adaptive Clinical Trial/ or multicenter study/ | 1245872 |
|  | 15 | randomized controlled trial.pt. | 615985 |
|  | 16 | clinical trial.pt. | 540075 |
|  | 17 | (clinical trial, phase i or clinical trial, phase ii or clinical trial, phase iii or clinical trial, phase iv).pt. | 84440 |
|  | 18 | (controlled clinical trial or multicenter study).pt. | 440867 |
|  | 19 | (clinical adj trial$).ti,ab,kf,kw. | 1764490 |
|  | 20 | ((singl$ or doubl$ or treb$ or tripl$) adj (blind$3 or mask$3)).ti,ab,kf. | 837229 |
|  | 21 | placebo$.ti,ab,kf. | 1017128 |
|  | 22 | (allocat$ adj2 random$).ti,ab,kf. | 164469 |
|  | 23 | (Randomi?ed adj2 trial$).ti,ab,kf. | 1550790 |
|  | 24 | rct.ti,ab,kf. | 139674 |
|  | 25 | (single arm adj3 (trial$ or stud$)).ti,ab,kf. | 36031 |
|  | 26 | (open label adj (trial$ or stud$)).ti,ab,kf. | 52999 |
|  | 27 | (non blinded adj (trial$ or stud$)).ti,ab,kf. | 905 |
|  | 28 | (pragmatic trial$ or pragmatic stud$).ti,ab,kf. or Pragmatic Clinical Trial/ or pragmatic trial/ | 13792 |
|  | 29 | or/4-28 | 6384853 |
| Observational studies | 30 | Epidemiologic Studies/ or epidemiology/ | 287693 |
|  | 31 | Observational Study/ | 536145 |
|  | 32 | Cohort Studies/ or cohort analysis/ | 1535181 |
|  | 33 | exp Case-Control Studies/ or exp case control study/ | 1781873 |
|  | 34 | Cross-Sectional Studies/ or cross-sectional study/ | 1158819 |
|  | 35 | Clinical Study/ | 173154 |
|  | 36 | Follow-Up Studies/ or follow up/ | 2979440 |
|  | 37 | Longitudinal Studies/ or longitudinal study/ | 397166 |
|  | 38 | Retrospective Studies/ or retrospective study/ | 2868742 |
|  | 39 | (Prospective Studies/ not Randomized Controlled Trials as Topic/) or (prospective study/ not "randomized controlled trial (topic)"/) | 1731907 |
|  | 40 | (observational adj (study or studies)).ti,ab,kf. | 475042 |
|  | 41 | (cohort adj (study or studies)).ti,ab,kf. | 896053 |
|  | 42 | cohort analy$.ti,ab,kf. | 36355 |
|  | 43 | case control.ti,ab,kf. | 387764 |
|  | 44 | cross sectional.ti,ab,kf. | 1327583 |
|  | 45 | (follow up adj (study or studies)).ti,ab,kf. | 147849 |
|  | 46 | longitudinal.ti,ab,kf. | 855908 |
|  | 47 | retrospective.ti,ab,kf. | 2209603 |
|  | 48 | (prospective adj (study or studies)).ti,ab,kf. | 591632 |
|  | 49 | (epidemiologic$ adj (study or studies)).ti,ab,kf. | 233408 |
|  | 50 | (evaluation adj (study or studies)).ti,ab,kf. | 20139 |
|  | 51 | (chart adj3 review$).ti,ab,kf. | 184930 |
|  | 52 | Or/30-51 | 11206342 |
| Exclusion terms | 53 | conference$.pt. | 6215960 |
|  | 54 | limit 53 to yr="1974-2022" | 5790426 |
|  | 55 | exp animals/ not exp humans/ | 10506517 |
|  | 56 | (comment or editorial or case reports or historical article).pt. | 5082068 |
|  | 57 | editorial/ or case report/ | 4476605 |
|  | 58 | (case stud$ or case report$).ti. | 913547 |
|  | 59 | or/54-58 | 23610255 |
| Combined | 60 | 3 and (29 or 52) | 10653 |
|  | 61 | 60 not 59 | 7334 |
|  | 62 | 3 use coch | 18 |
|  | 63 | 3 use cctz | 1298 |
|  | 64 | 63 not 59 | 990 |
|  | 65 | Or/61,62,64 | 7696 |
|  | 66 | limit 65 to yr="2023-current" | 1108 |
|  | 67 | remove duplicates from 66 | 799 |

Databases: EBM Reviews - Cochrane Central Register of Controlled Trials May 2024, EBM Reviews - Cochrane Database of Systematic Reviews 2005 to 12th June 2024, Embase 1974 to 18th June 2024, Ovid MEDLINE(R) Epub Ahead of Print and In-Process, In-Data-Review & Other Non-Indexed Citations and Daily 18th June 2024.

Abbreviations: PBC, primary biliary cholangitis; RCT, randomised controlled trial; SLR, systematic literature review.

**Table S 4****: Search terms used for congress websites in original SLR**

| **Congress** | **Link** | **Search strategy** | **Retrieved records 13.12.2022** | **Included records** |
| --- | --- | --- | --- | --- |
| AASLD Liver Meeting 2021 | <https://aasldpubs.onlinelibrary.wiley.com/toc/15273350/2021/74/S1> | Oral and poster abstracts in the 'PBC/PSC and Other Cholestatic Disease' category of the online platform were screened for relevance | 72 | 2 |
| AASLD Liver Meeting 2022 | <https://aasldpubs.onlinelibrary.wiley.com/toc/15273350/2022/76/S1> | Abstracts included in the abstract book under the 'PBC/PSC and Other Cholestatic Disease' category of the AASLD Liver Meeting 2022 Category Index were searched for and screened for relevance against the eligibility criteria | 59 | 5 |
| EASL International Liver Congress 2021 | <https://easl.eu/wp-content/uploads/2021/06/EASL_2021_Version-5-new.pdf> | The abstract book was searched for the following terms using the advanced search function in Adobe Acrobat:  Primary biliary cholangitis  Primary biliary cirrhosis  PBC  All retrieved abstracts were screened for relevance | 184 | 4 |
| EASL International Liver Congress 2022 | <https://easl.eu/wp-content/uploads/2022/06/EASL_2022_Version-4_Latest-version_compressed-1.pdf> | The abstract book was searched for the following terms using the advanced search function in Adobe Acrobat:  Primary biliary cholangitis  Primary biliary cirrhosis  PBC  All retrieved abstracts were screened for relevance | 324 | 1 |
| EASL Digital Liver Cancer Summit 2021 | <https://easl.eu/wp-content/uploads/2021/01/Digital-Liver-Cancer-Summit-2021-Abstract-book.pdf> | The abstract book was searched for the following terms using the advanced search function in Adobe Acrobat:  Primary biliary cholangitis  Primary biliary cirrhosis  PBC  All retrieved abstracts were screened for relevance | 0 | 0 |
| EASL Digital Liver Cancer Summit 2022 | <https://easl.eu/wp-content/uploads/2022/02/LCS-2022-Abstract-book-final.pdf> | The abstract book was searched for the following terms using the advanced search function in Adobe Acrobat:  Primary biliary cholangitis  Primary biliary cirrhosis  PBC  All retrieved abstracts were screened for relevance | 0 | 0 |

Footnote: Abstracts were not obtainable for the International Conference on Hepatology and Liver Disease or the World Conference of Gastroenterology and Hepatology.

Abbreviations: AASLD, American Association for the Study of Liver Diseases; EASL, European Association for the Study of the Liver; PBC, primary biliary cholangitis; PSC, primary sclerosing cholangitis; SLR, systematic literature review.

**Table S 5: Search terms used for congress websites in SLR Update I**

| **Congress** | **Link** | **Search strategy** | **Retrieved records 02.02.2024** | **Included records** |
| --- | --- | --- | --- | --- |
| AASLD Liver Meeting 2023 | <https://aasld.org/sites/default/files/2023-10/the_liver_meeting_boston_massachusetts_nov1_0.pdf> | The abstract book was searched for the following terms using the advanced search function in Adobe Acrobat:  Primary biliary cholangitis  Primary biliary cirrhosis  PBC  All retrieved abstracts were screened for relevance | 44 | 11 |
| AASLD Liver Meeting 2023 Late-breakers | <https://aasld.org/sites/default/files/2023-11/SLD-625%20Late%20Breaking%20Abstract.pdf> | The abstract book was searched for the following terms using the advanced search function in Adobe Acrobat:  Primary biliary cholangitis  Primary biliary cirrhosis  PBC  All retrieved abstracts were screened for relevance | 3 | 3 |
| EASL International Liver Congress 2023 | <https://easlcongress.eu/wp-content/uploads/2023/06/EASL_2023_Congress_Abstracts_version2_reduced_web-compressed-1.pdf> | The abstract book was searched for the following terms using the advanced search function in Adobe Acrobat:  Primary biliary cholangitis  Primary biliary cirrhosis  PBC  All retrieved abstracts were screened for relevance | 67 | 1 |
| EASL Digital Liver Cancer Summit 2023 | <https://easl.eu/wp-content/uploads/2023/04/EASL_Liver_Cancer_Summit_2023-Abstract-book-FINAL_15.04.2023-b.pdf> | The abstract book was searched for the following terms using the advanced search function in Adobe Acrobat:  Primary biliary cholangitis  Primary biliary cirrhosis  PBC  All retrieved abstracts were screened for relevance | 2 | 0 |

Footnote: Abstracts were not obtainable for the International Conference on Hepatology and Liver Disease or the World Conference of Gastroenterology and Hepatology.

Abbreviations: AASLD, American Association for the Study of Liver Diseases; EASL, European Association for the Study of the Liver; PBC, primary biliary cholangitis; PSC, primary sclerosing cholangitis; SLR, systematic literature review.

**Table S 6****: Search terms used for congress websites in SLR Update II**

| **Congress** | **Link** | **Search strategy** | **Retrieved records 22.07.24** | **Included records** |
| --- | --- | --- | --- | --- |
| AASLD Liver Meeting | N/A | N/A – No further meetings have been held since SLR Update I | N/A | N/A |
| EASL International Liver Congress 2024 | <https://www.easlcongress.eu/wp-content/uploads/2024/05/EASL_2024_Abstract_version1-1_27-05.pdf> | The abstract book was searched for the following terms using the advanced search function in Adobe Acrobat:  Primary biliary cholangitis  Primary biliary cirrhosis  PBC  All retrieved abstracts were screened for relevance | 93 | 22a |
| EASL Liver Cancer Summit 2023 | <https://easl.eu/wp-content/uploads/2024/02/EASL_Liver_Cancer_Summit_2024-Abstract-Book.pdf> | The abstract book was searched for the following terms using the advanced search function in Adobe Acrobat:  Primary biliary cholangitis  Primary biliary cirrhosis  PBC  All retrieved abstracts were screened for relevance | 0 | 0 |

Footnote: Abstracts were not obtainable for the International Conference on Hepatology and Liver Disease or the World Conference of Gastroenterology and Hepatology. ^a^Prior to evidence prioritisation.

Abbreviations: AASLD, American Association for the Study of Liver Diseases; EASL, European Association for the Study of the Liver; N/A, not applicable; PBC, primary biliary cholangitis; SLR, systematic literature review.

**Table S 7****: Search terms used for ClinicalTrials.gov**

| **SLR update (Date)** | **Condition** | **Phases** | **Study results** | **Recruitment status** | **Results posted date** | **Retrieved records** | **Included records** |
| --- | --- | --- | --- | --- | --- | --- | --- |
| Original SLR (19.12.2022) | "primary biliary cholangitis" OR "primary biliary cirrhosis" OR "primary biliary cholestasis" OR "PBC" | Any | "Studies With Results" | All | First posted: Any time | 25 | 6 |
| SLR Update I (05.12.2023) |  |  |  |  | Last update posted: November 2022–onwards | 1 | 1 |
| SLR Update II (12.07.2024) |  |  |  |  | Last update posted: December 2023–onwards | 2 | 1a |

Footnote: ^a^Prior to evidence prioritisation.

Abbreviations: PBC, primary biliary cholangitis; SLR, systematic literature review.

## NMA eligibility criteria

The eligibility criteria for the NMA is presented in **Table S 8**.

**Table S 8****: Eligibility criteria used for included publications in NMA**

| **Study characteristics** | **Inclusion** | **Exclusion** |
| --- | --- | --- |
| **P**opulation | - Unselected adult patients (aged ≥18 years) with PBC who had had an inadequate response to or unacceptable side effects with UDCA | - Patients without PBC - Patients who had not been exposed to UDCA - Patients who were able to tolerate UDCA and had responded adequately - Children or adolescents (<18 years old) |
| **I**ntervention/ **C**omparator | - Elafibranor 80 mg - Seladelpar 10 mg | - Studies not including elafibranor 80 mg or seladelpar 10 mg |
| **O**utcomes | Outcomes reported after 12 months of treatment including:   - Clinical efficacy/effectiveness outcomes, including but not limited to:   1. Survival outcomes   2. Response rates   3. Biochemical responses (e.g., CFB in ALP levels, etc.) - Pruritus outcomes, including but not limited to:   1. Patients experiencing pruritus   2. Pruritus-specific health-related QoL outcome measures such as:   3. ItchyQoL - Safety outcomes, including but not limited to:   1. Adverse events (AEs) (including TRAEs and serious AEs)   2. Deaths   3. Discontinuation due to AEs/TRAEs | - Studies not reporting any relevant outcomes after 12 months of treatment - Studies not reporting any relevant outcomes, e.g., pharmacokinetics. - Studies reporting a relevant outcome but in a mixed population where results for PBC are not reported separately. |
| **S**tudy Design | - RCTs | - Non-randomised interventional studies (e.g., single-arm trials) - Observational studies - Non-primary research publications, including: - Narrative reviews - Editorials - Guidelines - Commentaries - Opinion pieces |

Abbreviations: AE – adverse event; ALP – alkaline phosphatase; NMA – network meta-analysis; PBC – Primary Biliary Cholangitis; QoL – quality of life; TRAE – treatment related adverse event; UDCA – ursodeoxycholic acid

## Method to align ELATIVE trial data to RESPONSE

Following the feasibility assessment, only ELATIVE and RESPONSE were retained, but differences were identified between them in the upper limit of normal (ULN) definitions for alkaline phosphatase (ALP) and total bilirubin (TB) used in their eligibility criteria.^1,2^ Moreover, the ULN for ALP and TB were used in the definition of biochemical response and ALP normalisation, leading to discrepancies between the two studies in the definitions of these endpoints.^3,4^

To align the trial populations and outcomes in ELATIVE with those from RESPONSE, the following criteria from RESPONSE were applied to the ELATIVE patient level data (PLD) at baseline to exclude patients in ELATIVE who would not have been eligible for enrolment in RESPONSE:

- ALP ≥1.67 x ULN (defined as 1.67 x 116 U/L)
- TB ≤ 2x ULN (defined as 2 x 1.1 mg/dL)

Prior to conducting the ITC, all outcomes in ELATIVE were re-estimated using the truncated ELATIVE population (n=145). For the binary endpoints, the number of responders were recalculated, applying the ALP and TB ULN definitions used in RESPONSE to determine cholestasis response and ALP normalisation. Estimates for the continuous outcomes were derived using the same methodology as originally reported for ELATIVE, which was the estimation of the least-square mean (LSM) change from baseline.^3^ The analysis used the mixed model for repeated measures (MMRM) with fixed effects for treatment, baseline values and the stratification factors, scheduled visits (until week 52) and the interaction between treatment arm and visit date were adjusted for. Random-effects models used trial-specific baselines to account for inter-subject variability, incorporating random intercepts for individual participants to capture deviations from the overall trajectory, ensuring robust estimation of treatment effects.

## NMA analysis methodology

For continuous outcomes, the mean change from baseline in ELATIVE and RESPONSE was assumed to follow a normal distribution. Relative differences in change from baseline between intervention and placebo in each study were synthesised to estimate the relative difference in change from baseline between elafibranor and seladelpar.

For binary outcomes, the event rates were assumed to follow a binomial distribution. The odds ratios of outcomes for interventions compared to placebo in each study were synthesised to estimate the odds ratios of elafibranor compared to seladelpar.

A Markov chain Monte Carlo approach was used for inference, enabling the estimation of posterior distributions for the parameters of interest.^5^ Vague priors of $N(0,{100}^{2})$ were used for treatment effects and baseline study effects to allow for a broad range of plausible values, minimising the risk of bias by ensuring that the derived treatment effects are primarily driven by data rather than the choice of prior.

Informative priors were used for between-study standard deviation (SD). The use of informative priors ensures that the model reflects existing knowledge while still allowing for variability across studies, in addition to helping mitigate the risk of overfitting in complex models, particularly when analysing sparse or heterogenous data. This enables more reliable inferences to be made about treatment effects.^6^ The informative priors were truncated, conservatively assuming that the OR for outcomes between treatments could not exceed 10.^7^ Truncation limits the parameter space to clinically plausible values, preventing the model from considering extreme or unrealistic treatment effects.^7^ In order to truncate the priors for continuous outcomes, the conversion of between-study SD to the mean difference scale was informed by Ren et al. (2018).^7^

Convergence to the target posterior distributions were assessed using the Brooks-Gelman-Rubin (BGR) plots.^8^ To estimate parameters, a suitable burn-in was selected, and thinning was allowed, if required. Burn-in length was determined by examining the BGR plots and ensuring that all chains had mixed well and converged before proceeding with the analysis, ensuring that subsequent samples used for parameter estimation are representative of the target posterior distribution.^9,10^

Both random-effects and fixed-effect models were assessed, with the fixed-effect results considered as the base-case instead of the random-effects model only if the residual deviance is at least three points lower in the fixed-effect model than the random-effects model.^11^ Random-effects models are generally preferred as they account for heterogeneity in treatment effects between studies; heterogeneity is expected between studies in the network.^12^ Conversely, fixed-effect models make the unrealistic assumption that treatment effects are identical across studies, ignoring potential heterogeneity.^13^

The relative effect matrices, showing pairwise comparisons between elafibranor, seladelpar and placebo, were produced and are reported in Section S9.

## Studies excluded from the NMA

Of the 43 unique studies extracted in the SLR, there were two elafibranor studies (Schattenberg [2021] and ELATIVE) and five seladelpar studies (Jones 2017, Bowlus 2022, Bowlus and Choi 2022, ENHANCE, and RESPONSE).^1, 4,14–18^

During the feasibility assessment, the studies were reviewed to identify potential connected networks of trials. Given that the ELATIVE study was designed to evaluate efficacy after 52 weeks of treatment, studies which assessed fewer than 12 months of treatment were excluded in line with the NMA eligibility criteria (Table S 8; Section S2).^3^ Therefore, Jones 2017, Schattenberg 2021 and ENHANCE were excluded.^15,17,18^ The Bowlus 2022 study included study arms of seladelpar at doses of 2 mg, 5 mg and 10 mg per day without a placebo-control arm,^16^ and so did not provide evidence for seladelpar compared to another comparator in the network of evidence (i.e., placebo or elafibranor). Therefore, Bowlus 2022 could not provide a feedback loop for comparisons between elafibranor and seladelpar, and so was excluded. The Bowlus and Choi 2022 study included pooled data from Bowlus 2022 and ENHANCE to fill evidence gaps in ENHANCE, and was excluded due to the studies it reported on having already been excluded.^14^

A summary of the 41 studies not reporting on elafibranor or seladelpar is presented in Table S 9Table S 9.

**Table S 9****: Summary of studies identified from the SLR but excluded from the NMA and their reason for exclusion**

| **Study name** | **Reference** | **Reason for exclusion** |
| --- | --- | --- |
| 1. Askari 2010 | Askari F, Innis D, Dick RB, *et al.* Treatment of primary biliary cirrhosis with tetrathiomolybdate: results of a double-blind trial. Translational Research: The Journal Of Laboratory & Clinical Medicine 2010;155:123-30. | Not a comparator of interest |
| 2. BEZURSO (NCT01654731) | Corpechot C, Chazouilleres O, Rousseau A, *et al.* A Placebo-Controlled Trial of Bezafibrate in Primary Biliary Cholangitis. New England Journal of Medicine 2018;378:2171-2181. | Not a comparator of interest |
|  | Corpechot C, Rousseau A, Lemoinne S, *et al.* Bezafibrate add-on therapy in high-risk primary biliary cholangitis is associated with an improvement of fibrometer and fibrometer-VCTE, two high-accuracy non-invasive fibrosis tests extensively validated in frequent chronic liver diseases. Journal of hepatology 2019;70:387‐. |  |
|  | Kremer AE, Le Cleac HA, Lemoinne S, *et al.* Antipruritic effect of bezafibrate and serum autotaxin measures in patients with primary biliary cholangitis. Gut. 2018. |  |
|  | Nct. Phase 3 Study of Bezafibrate in Combination With Ursodeoxycholic Acid in Primary Biliary Cirrhosis. https://clinicaltrials.gov/show/NCT01654731 2012. |  |
| 3. Li 2022 (ChiCTR1800020160) | Li C, Zheng K, Chen Y, *et al.* A randomized, controlled trial on fenofibrate in primary biliary cholangitis patients with incomplete response to ursodeoxycholic acid. Therapeutic Advances in Chronic Disease 2022;13:20406223221114198. | Not a comparator of interest |
|  | ChiCtr. Clinical research for fenofibrate in the treatment of refractory primary biliary cirrhosis. https://trialsearch.who.int/Trial2.aspx?TrialID=ChiCTR1800020160 2018. |  |
| 4. COBALT | Kowdley, KV; Brookhart, MA; Hirschfield, G *et al.* Efficacy of obeticholic acid (oca) vs placebo and external control (EC) on clinical outcomes in primary biliary cholangitis (PBC). Hepatology (Baltimore, Md). 2023;77(5):E144‐E146. | Not a comparator of interest |
|  | ClinicalTrialsgov. Phase 4 Study of Obeticholic Acid Evaluating Clinical Outcomes in Patients With Primary Biliary Cholangitis. 2023. Available from: https://ClinicalTrials.gov/show/NCT02308111. |  |
|  | Kowdley, K; Hirschfield, G; Mayne, T *et al.* 4538-C \| Clinical trial and real-world outcomes in patients with primary biliary cholangitis treated with obeticholic acid per current US label. 2023. AASLD 2023; |  |
|  | Kowdley, K; Mayne, T; Wheeler, D *et al.* 4578-C \| The challenge of confirmatory trials in rare disease: Lessons learned from the cobalt trial in primary biliary cholangitis 2023. AASLD 2023; |  |
| 5. Combes 2005 | Combes B, Emerson SS, Flye NL, *et al.* Methotrexate (MTX) plus ursodeoxycholic acid (UDCA) in the treatment of primary biliary cirrhosis. Hepatology 2005;42:1184-93. | Not a comparator of interest |
| 6. ENHANCE (NCT03602560) | Hirschfield, GM; Shiffman, ML; Gulamhusein, A *et al.* Seladelpar efficacy and safety at 3 months in patients with primary biliary cholangitis: ENHANCE, a phase 3, randomized, placebo-controlled study. Hepatology. 2023;78(2):397-415. | Assessed fewer than 12 months of treatment |
|  | Nct. Seladelpar in Subjects With Primary Biliary Cholangitis (PBC) and an Inadequate Response to or an Intolerance to Ursodeoxycholic Acid (UDCA). https://clinicaltrials.gov/show/NCT03602560 2018. |  |
|  | Anonymous. ENHANCE: Safety and Efficacy of Seladelpar in Patients With Primary Biliary Cholangitis-A Phase 3, International, Randomized, Placebo-Controlled Study. Gastroenterology & Hepatology 2021;17:5-6. |  |
|  | Choi Y-J, Johnson JD, Schwab A, *et al.* Seladelpar, a PPAR-delta agonist, improves inflammatory lipid mediators in the serum metabolome in patients with primary biliary cholangitis (PBC), in Hepatology, Wiley 111 River St, Hoboken 07030-5774, NJ USA, 2022. |  |
| 7. FITCH (NCT02701166) | de Vries E, Bolier R, Goet J, *et al.* Fibrates for Itch (FITCH) in Fibrosing Cholangiopathies: a Double-Blind, Randomized, Placebo-Controlled Trial. Gastroenterology 2021;160:734‐743.e6. | Not a comparator of interest |
|  | Euctr NL. The effect of bezafibrate on itch in a subset of liver diseases. https://trialsearch.who.int/Trial2.aspx?TrialID=EUCTR2014-001438-27-NL 2015. |  |
|  | Nct. The Effect of Bezafibrate on Cholestatic Itch. https://clinicaltrials.gov/show/NCT02701166 2016. |  |
| 8. GLIMMER (NCT02966834) | Levy C, Kendrick S, Bowlus CL, *et al.* GLIMMER: A randomized Phase 2b dose-ranging trial of linerixibat in primary biliary cholangitis patients with pruritus. Clinical gastroenterology and hepatology : the official clinical practice journal of the American Gastroenterological Association. 2022;04. | Not a comparator of interest |
|  | Carreño F, Mehta R, Ribeiro A, *et al.* Linerixibat dose-response analysis of C4 concentrations as a quantitative approach to predict gastrointestinal tolerability. Journal of Hepatology 2022:S328-S329. |  |
|  | Euctr ES. Dose response study of GSK2330672 for the treatment of pruritus in patients with primary biliary cholangitis – The GLIMMER Study. https://trialsearch.who.int/Trial2.aspx?TrialID=EUCTR2016-002416-41-ES 2016. |  |
|  | Fettiplace, J; Swift, B; Zhang, S *et al.* Investigation of Linerixibat 40 Mg BID for Cholestatic Pruritus of Primary Biliary Cholangitis; Further Data From the Phase 2b GLIMMER Study to Support the Phase 3 GLISTEN Study. American Journal of Gastroenterology. 2022;117(10 Supplement 2):S16. |  |
|  | Fettiplace J, Swift B, Zhang S, *et al.* Investigation of linerixibat 40mg BID for cholestatic pruritus of primary biliary cholangitis (PBC); further data from the phase 2b GLIMMER study to support the phase 3 GLISTEN study. Journal of Hepatology 2022;77(Supplement 1):S335-S336. |  |
|  | Jia J, Jones D, Maltzahn RV, *et al.* Improvement in itch correlates with improved sleep in GLIMMER, a Phase 2b trial of linerixibat for the treatment of cholestatic pruritus in primary biliary cholangitis (PBC). Hepatology international 2022;16:S297‐S298. |  |
|  | Jia J, Levy C, Kendrick S, *et al.* GLIMMER: a randomized double-blind placebo-controlled study of linerixibat, an ileal bile acid transporter inhibitor, in the treatment of cholestatic pruritus in primary biliary cholangitis (PBC). Hepatology international 2022;16:S296‐S297. |  |
|  | Jones DE, Maltzahn RV, Smith H, *et al.* 5-D Itch and Quality of Life in Pbc Patients with Cholestatic Pruritus from the Phase 2b Study Glimmer. Gastroenterology 2022;162(7 Supplement):S-1285-S-1286. |  |
|  | Nct. Dose Response Study of GSK2330672 for the Treatment of Pruritus in Patients With Primary Biliary Cholangitis. https://clinicaltrials.gov/show/NCT02966834 2016. |  |
|  | Smith H, Fettiplace J, von Maltzahn R, *et al.* More than just an itch: impact of cholestatic pruritus in primary biliary cholangitis (PBC) on health-related quality of life (HRQoL). Journal of Hepatology 2022;77(Supplement 1):S327-S328. |  |
|  | Tanaka, A; Atsukawa, M; Tsuji, K *et al.* Japanese subgroup analysis of GLIMMER: A global Phase IIb study of linerixibat for the treatment of cholestatic pruritus in patients with primary biliary cholangitis. Hepatology Research. 2023;53(7):629-640. |  |
|  | Tanaka, A; Suyama, A; Ito, H *et al.* Investigation of linerixibat 40 mg BID (twice daily) for cholestatic pruritus of primary biliary cholangitis (PBC) in the glimmer phase 2b study; further analysis of the Japanese subgroup. Hepatology International. 2023;17(Supplement 1):S185-S186. |  |
| 9. Gonzalez-Koch 1997 | Gonzalez-Koch A, Brahm J, Antezana C, *et al.* The combination of ursodeoxycholic acid and methotrexate for primary biliary cirrhosis is not better than ursodeoxycholic acid alone. Journal of Hepatology 1997;27:143-9. | Not a comparator of interest |
| 10. Hejda 2023 | Hejda, V; Louvet, A; Civitarese, A *et al.* Results from a planned interim analysis of a randomized, double-blind, active-controlled trial evaluating the effects of obeticholic acid and bezafibrate on serum biomarkers in primary biliary cholangitis. Journal of Hepatology. 2023;78(Supplement 1):S45. | Not a comparator of interest |
| 11. Hosonuma 2015 | Hosonuma K, Sato K, Yamazaki Y, *et al.* A prospective randomized controlled study of long-term combination therapy using ursodeoxycholic acid and bezafibrate in patients with primary biliary cirrhosis and dyslipidemia. American Journal of Gastroenterology 2015;110:423-31. | Not a comparator of interest |
| 12. Ikeda 1996 | Ikeda T, Tozuka S, Noguchi O, *et al.* Effects of additional administration of colchicine in ursodeoxycholic acid-treated patients with primary biliary cirrhosis: a prospective randomized study. Journal of Hepatology 1996;24:88-94. | Not a comparator of interest |
| 13. Iwasaki 2008 | Iwasaki S, Ohira H, Nishiguchi S, *et al.* The efficacy of ursodeoxycholic acid and bezafibrate combination therapy for primary biliary cirrhosis: a prospective, multicenter study. Hepatology research 2008;38:557‐564. | Not a comparator of interest |
| 14. Kanda 2003 | Kanda T, Yokosuka O, Imazeki F, *et al.* Bezafibrate treatment: a new medical approach for PBC patients? Journal of Gastroenterology 2003;38:573-8. | Not a comparator of interest |
| 15. Kurihara 2000 | Kurihara T, Niimi A, Maeda A, *et al.* Bezafibrate in the treatment of primary biliary cirrhosis: comparison with ursodeoxycholic acid. American Journal of Gastroenterology 2000;95:2990-2. | Not a comparator of interest |
| 16. Levy 2023 (Study 213, Study 214) | Levy C.; Hejda V.; Louvet A. *et al.* 5019-C \| Combined effect of obeticholic acid and bezafibrate in patients with primary biliary cholangitis and inadequate response to or intolerance of ursodeoxycholic acid: results from two phase 2 clinical trials 2023. AASLD 2023; | Not a comparator of interest |
| 17. Liberopoulos 2010 | Liberopoulos EN, Florentin M, Elisaf MS, *et al.* Fenofibrate in primary biliary cirrhosis: a pilot study. Open cardiovascular medicine journal 2010;4:120‐126. | Not a comparator of interest |
| 18. Lim 1997 | Lim AG, Wolfhagen FH, Verma A, *et al.* Soluble intercellular adhesion molecule-1 in primary biliary cirrhosis: effect of ursodeoxycholic acid and immunosuppressive therapy. European Journal of Gastroenterology & Hepatology 1997;9:155-61. | Not a comparator of interest |
| 19. Nakai 2000 | Nakai S, Masaki T, Kurokohchi K, *et al.* Combination therapy of bezafibrate and ursodeoxycholic acid in primary biliary cirrhosis: a preliminary study. Official journal of the American College of Gastroenterology\| ACG 2000;95:326-327. | Not a comparator of interest |
| 20. Hirschfield 2015 (NCT00550862) | Hirschfield GM, Mason A, Luketic V, *et al.* Efficacy of obeticholic acid in patients with primary biliary cirrhosis and inadequate response to ursodeoxycholic acid. Gastroenterology 2015;148:751‐61.e8. | Not a comparator of interest |
|  | Nct. Study of INT 747 in Combination With URSO in Patients With Primay Biliary Cirrhosis (PBC). https://clinicaltrials.gov/show/NCT00550862 2007. |  |
| 22. Hirschfield 2021 (NCT00746486) | Hirschfield GM, Beuers U, Kupcinskas L, *et al.* A placebo-controlled randomised trial of budesonide for PBC following an insufficient response to UDCA. Journal of Hepatology 2021;74:321-329. | Not a comparator of interest |
|  | Euctr ES. Double-blind, randomised, placebo-controlled, multi-centre phase III clinical study comparing the combination of ursodeoxycholic acid capsules plus budesonide capsules to ursodeoxycholic acid capsules plus placebo in the treatment of primary biliary cirrhosis - Ursodeoxycholic acid plus budesonide vs. ursodeoxycholic acid alone in PBC. https://trialsearch.who.int/Trial2.aspx?TrialID=EUCTR2007-004040-70-ES 2008. |  |
|  | Nct. Ursodeoxycholic Acid Plus Budesonide Versus Ursodeoxycholic Acid Alone in Primary Biliary Cirrhosis (PBC). https://clinicaltrials.gov/show/NCT00746486 2008. |  |
| 23. HAART (NCT01614405) | Lytvyak E, Hosamani I, Montano-Loza AJ, *et al.* Randomized clinical trial: Combination antiretroviral therapy with tenofovir-emtricitabine and lopinavir-ritonavir in patients with primary biliary cholangitis. Canadian Liver Journal 2019;2:31-44 | Not a comparator of interest |
|  | Nct. Highly Active Antiretroviral Therapy for Patients With Primary Biliary Cirrhosis. https://clinicaltrials.gov/show/NCT01614405 2011. |  |
| 24. Hegade 2017 (NCT01899703) | Hegade VS, Kendrick SF, Dobbins RL, *et al.* Effect of ileal bile acid transporter inhibitor GSK2330672 on pruritus in primary biliary cholangitis: a double-blind, randomised, placebo-controlled, crossover, phase 2a study. Lancet 2017;389:1114-1123. | Not a comparator of interest |
|  | Hegade VS, Kendrick SF, Dobbins RL, *et al.* BAT117213: Ileal bile acid transporter (IBAT) inhibition as a treatment for pruritus in primary biliary cirrhosis: study protocol for a randomised controlled trial. BMC Gastroenterology 2016;16:71. |  |
|  | Nct. A Study to Evaluate the Safety, Tolerability, Pharmacokinetics (PK) and Pharmacodynamics (PD) of Repeat Doses of GSK2330672 Administration in Subjects With Primary Biliary Cirrhosis (PBC) and Symptoms of Pruritus. https://clinicaltrials.gov/show/NCT01899703 2013. |  |
| 25. CLARITY (NCT01904058) | Nct. Phase 2 Study to Evaluate LUM001 in Combination With Ursodeoxycholic Acid in Patients With Primary Biliary Cirrhosis. https://clinicaltrials.gov/show/NCT01904058 2013. | Not a comparator of interest |
| 26. Mayo 2018 (NCT02026401) | Mayo MJ, Wigg AJ, Leggett BA, *et al.* NGM282 for Treatment of Patients With Primary Biliary Cholangitis: A Multicenter, Randomized, Double-Blind, Placebo-Controlled Trial. Hepatology Communications 2018;2:1037-1050 | Not a comparator of interest |
|  | Nct. Phase 2 Study of NGM282 in Patients With Primary Biliary Cirrhosis. https://clinicaltrials.gov/show/NCT02026401 2013. |  |
| 27. Schramm 2022 (NCT02516605) | Schramm C, Wedemeyer H, Mason A, *et al.* Farnesoid X receptor agonist tropifexor attenuates cholestasis in a randomised trial in patients with primary biliary cholangitis. JHEP Reports : Innovation in Hepatology / EASL 2022;4:100544. | Not a comparator of interest |
|  | Nct. A Multi-part, Double Blind Study to Assess Safety, Tolerability and Efficacy of Tropifexor (LJN452) in PBC Patients. https://clinicaltrials.gov/show/NCT02516605 2015. |  |
| 28. Jones 2017 (NCT02609048) | Jones D, Boudes PF, Swain MG, *et al.* Seladelpar (MBX-8025), a selective PPAR-delta agonist, in patients with primary biliary cholangitis with an inadequate response to ursodeoxycholic acid: a double-blind, randomised, placebo-controlled, phase 2, proof-of-concept study. The lancet gastroenterology and hepatology 2017;2:716‐726. | Assessed fewer than 12 months of treatment |
| 29. NCT02943447 | Nct. Safety, Tolerability, and Efficacy of GS 9674 in Adults With Primary Biliary Cholangitis Without Cirrhosis. https://clinicaltrials.gov/show/NCT02943447 2016. | Not a comparator of interest |
| 30. Bowlus 2022 (NCT02955602) | Bowlus CL, Galambos MR, Aspinall RJ, *et al.* A phase II, randomized, open-label, 52-week study of seladelpar in patients with primary biliary cholangitis. Journal of hepatology 2022;77:353‐364. | Could not provide a feedback loop for analyses |
|  | Bowlus CL, Neff GW, Aspinall R, *et al.* Efficacy and safety of seladelpar, a selective peroxisome proliferator-activated receptor delta agonist, in primary biliary cholangitis: 52-week analysis of an ongoing international, randomized, dose ranging phase 2 study. Hepatology (Baltimore, Md.) 2018;68:1446A‐1447A. |  |
|  | Hansen B, Watkins E, Yang K, *et al.* Seladelpar treatment of patients with primary biliary cholangitis (PBC) for 2 years improves the GLOBE PBC score and predicts improved transplant-free survival. Journal of Hepatology 2022;77(Supplement 1):S322-S323. |  |
|  | Kremer AE, Mayo MJ, Hirschfield G, *et al.* Seladelpar improved measures of pruritus, sleep, and fatigue and decreased serum bile acids in patients with primary biliary cholangitis. Liver International 2022;42(1):112-123. |  |
|  | Mayo MJ, Vierling JM, Bowlus CL, *et al.* Long-term safety and efficacy of seladelpar in patients with primary biliary cholangitis (pbc): 2-year results from a long-term study. Hepatology (Baltimore, Md.) 2021;74:71A‐73A. |  |
|  | Nct. Seladelpar (MBX-8025) in Subjects With Primary Biliary Cholangitis (PBC). https://clinicaltrials.gov/show/NCT02955602 2016. |  |
| 31. Bowlus and Choi:NCT02955602; ENHANCE (NCT03602560) | Bowlus CL, Choi Y-J, Yang K, *et al.* Seladelpar improved the lipid profile of patients with primary biliary cholangitis (pbc): results from phase 2 and 3 clinical studies, In Hepatology, WILEY 111 River St, Hoboken 07030-5774, NJ USA, 2022. | Comprised of studies which had already been excluded |
|  | Gulamhusein A, Neff G, Goel A, *et al.* Treatment with seladelpar in patients with primary biliary cholangitis (PBC) and prior experience with obeticholic acid (OCA) or fibrates. J Hepatol 2021;75:S690-S691. |  |
| 32. Vuppalanchi 2022 (NCT03112681) | Vuppalanchi R, Caldwell SH, Pyrsopoulos N, *et al.* Proof-of-concept study to evaluate the safety and efficacy of saroglitazar in patients with primary biliary cholangitis. Journal of hepatology 2022;76:75‐85.. | Not a comparator of interest |
|  | Nct. A Study to Evaluate Safety, Tolerability and Efficacy of Saroglitazar Magnesium in Patients With Primary Biliary Cholangitis (EPICS). https://clinicaltrials.gov/show/NCT03112681 2017. |  |
| 33. Schattenberg 2021 (NCT03124108) | Schattenberg JM, Pares A, Kowdley KV, *et al.* A randomized placebo-controlled trial of elafibranor in patients with primary biliary cholangitis and incomplete response to UDCA. Journal of hepatology 2021;74:1344‐1354. | Assessed fewer than 12 months of treatment |
|  | Euctr ES. Phase 2 Efficacy & Safety Study of Elafibranor in patients with PBC. https://trialsearch.who.int/Trial2.aspx?TrialID=EUCTR2016-003817-80-ES 2017. |  |
|  | Nct. Study to Evaluate the Efficacy and Safety of Elafibranor in Patients With Primary Biliary Cholangitis (PBC) and Inadequate Response to Ursodeoxycholic Acid. https://clinicaltrials.gov/show/NCT03124108 2017. |  |
| 34. NCT03226067 | Invernizzi, P; Carbone, M; Jones, D *et al.* Setanaxib, a first-in-class selective NADPH oxidase 1/4 inhibitor for primary biliary cholangitis: A randomized, placebo-controlled, phase 2 trial. Liver International. 2023;43(7):1507-1522. | Not a comparator of interest |
|  | NCT. Study to Assess Safety & Efficacy of GKT137831 in Patients With Primary Biliary Cholangitis Receiving Ursodiol. 2017. |  |
|  | Euctr BE. A study assessing the efficacy and safety of GKT137831 in Patients with Primary Biliary Cholangitis Receiving Ursodeoxycholic Acid and with Persistently Elevated Alkaline Phosphatase. https://trialsearch.who.int/Trial2.aspx?TrialID=EUCTR2016-004599-23-BE 2017. |  |
|  | Jones, D; Carbone, M; Invernizzi, P *et al.* Impact of setanaxib on quality of life outcomes in primary biliary cholangitis in a phase 2 randomized controlled trial. Hepatology communications. 2023;7(3):e0057. |  |
|  | Jones D, Carbone M, Invernizzi P, *et al.* Quality of life outcomes in patients with primary biliary cholangitis treated with setanaxib: post-hoc results from a phase 2 randomised, placebo-controlled trial. Journal of Hepatology 2022;77(Supplement 1):S94-S95. |  |
|  | Levy C, Carbone M, Wiesel P, *et al.* Setanaxib reduces cholestasis and fatigue in patients with primary biliary cholangitis and liver stiffness >=9.6 kpa: Post-hoc analyses from a randomized, controlled, phase 2 trial. Hepatology 2021;74(SUPPL 1):782A-783A. |  |
| 35. Xiang 2021 (NCT03345589) | Xiang X, Yang X, Shen M, *et al.* Ursodeoxycholic Acid at 18-22 mg/kg/d Showed a Promising Capacity for Treating Refractory Primary Biliary Cholangitis. Canadian Journal of Gastroenterology & Hepatology 2021;2021:6691425. | Not a comparator of interest |
|  | Nct. A Trial of 18-22mg/kg/d Ursodeoxycholic in Refractory Primary Biliary Cholangitis. https://clinicaltrials.gov/show/NCT03345589 2017. |  |
| 36. NCT03394924 | Nct. A Study to Assess the Safety, Tolerability, Pharmacokinetics and Efficacy of EDP-305 in Subjects With Primary Biliary Cholangitis. https://clinicaltrials.gov/show/NCT03394924 2018. | Not a comparator of interest |
| 37. NCT03633227 | Nct. Study of OCA Evaluating Pharmacokinetics and Safety in Patients With PBC and Hepatic Impairment. https://clinicaltrials.gov/show/NCT03633227 2018. | Not a comparator of interest |
| 38. POISE (NCT01473524) | Nevens F, Andreone P, Mazzella G, *et al.* A Placebo-Controlled Trial of Obeticholic Acid in Primary Biliary Cholangitis. New England journal of medicine 2016;375:631‐643. | Not a comparator of interest |
|  | Andreone P, Floreani A, Invernizzi P, *et al.* Durable response in the markers of cholestasis through 24 months of open-label extension with obeticholic acid in Italian patients with primary biliary cholangitis. Digestive and liver disease 2017;49:e21‐. |  |
|  | Andreone P, Floreani A, Invernizzi P, *et al.* Durable response in the markers of cholestasis through 36 months of open-label extension with obeticholic acid in Italian patients with primary biliary cholangitis. Digestive and liver disease 2018;50:26‐. |  |
|  | Bonder A.; Wheeler, D; Nair, R *et al.* 4545-C \| Effect of obeticholic acid on prognostic thresholds of gamma-glutamyl transferase and alkaline phosphatase levels: sub-analysis of the phase 3 poise trial in primary biliary cholangitis 2023. AASLD 2023; |  |
|  | Bowlus C, Trauner M, Liberman A, *et al.* Long-Term Efficacy and Safety of Obeticholic Acid in Patients with PBC from POISE Grouped by Risk of Disease Progression. Zeitschrift fur Gastroenterologie 2021;59(1):e14-e15. |  |
|  | Bowlus CL, Pockros PJ, Kremer AE, *et al.* Three years of Obeticholic Acid (OCA) Therapy Results in Histological Improvements in Patients with Primary Biliary Cholangitis: further Analysis of the POISE Biopsy Substudy. Zeitschrift fur Gastroenterologie 2019;57:e9‐. |  |
|  | Bowlus CL, Pockros PJ, Kremer AE, *et al.* Long-Term Obeticholic Acid Therapy Improves Histological Endpoints in Patients With Primary Biliary Cholangitis. Clinical gastroenterology and hepatology 2020;18:1170‐1178.e6. |  |
|  | Bowlus CL, Trauner M, Liberman A, *et al.* Long-term efficacy and safety of obeticholic acid in patients with PBC from the POISE trial grouped biochemically by risk of disease progression. Digestive and Liver Disease 2021;53(Supplement 1):S18. |  |
|  | Carbone M, Harms MH, Lammers WJ, *et al.* Clinical application of the GLOBE and United Kingdom-primary biliary cholangitis risk scores in a trial cohort of patients with primary biliary cholangitis. Hepatology Communications 2018;2:683-692. |  |
|  | Floreani A, Bowlus CL, Trauner M, *et al.* Long-Term Efficacy And Safety Of Obeticholic Acid In Patients With Pbc From The Poise Trial Grouped Biochemically By Risk Of Disease Progression. Digestive and Liver Disease 2021;53(Supplement 3):S109. |  |
|  | Halilbasic E, Hofer H, Munda P, *et al.* Durable response in the markers of cholestasis through 18 months of open-label extension with obeticholic acid in Austrian and German patients with primary biliary cholangitis. Zeitschrift fur Gastroenterologie 2017;55. |  |
|  | Halilbasic E, Zoller H, Munda P, *et al.* Durable response in the markers of cholestasis through 36 months of open-label extension with obeticholic acid in Austrian and german patients with primary biliary cholangitis. Zeitschrift fur Gastroenterologie 2018;56:e47‐. |  |
|  | Harms MH, Hirschfield GM, Floreani A, *et al.* Obeticholic acid is associated with improvements in AST-to-platelet ratio index and GLOBE score in patients with primary biliary cholangitis. JHEP Reports : Innovation in Hepatology / EASL 2021;3:100191. |  |
|  | Hirschfield G, Carbone M, Jones D, *et al.* Durability of obeticholic acid response in PBC patients who did not achieve poise trial criteria. Gut 2021;70(SUPPL 1):A13. |  |
|  | Hirschfield G, Jones D, Carbone M, *et al.* Long-Term Efficacy and Safety of Obetichoclic Acid in Primary Biliary Cholangitis: Responder Analysis of over 5 Years of Treatment in the Poise Trial. Journal of the Canadian Association of Gastroenterology. Conference: Canadian Digestive Diseases Week, CDDW 2021;4. |  |
|  | Jones D, Carbone M, Mells G, *et al.* Predicted risk of end stage liver disease utilizing the UK-PBC risk score in PBC patients. Gut 2021;70(SUPPL 1):A153-A154. |  |
|  | Kremer A, Hirschfield G, Jones D, *et al.* Pruritus Experience in Patients with PBC Treated with Obeticholic Acid Through 6 Years: Patient-Reported Quality of Life. Zeitschrift fur Gastroenterologie 2021;59(1):e22. |  |
|  | Nct. Phase 3 Study of Obeticholic Acid in Patients With Primary Biliary Cirrhosis. https://clinicaltrials.gov/show/NCT01473524 2011. |  |
|  | Pares A, Shiffman M, Vargas V, *et al.* Reduction and stabilization of bilirubin with obeticholic acid treatment in patients with primary biliary cholangitis. Liver International 2020;40:1121-1129. |  |
|  | Trauner M, Nevens F, Shiffman ML, *et al.* Long-term efficacy and safety of obeticholic acid for patients with primary biliary cholangitis: 3-year results of an international open-label extension study. The lancet. Gastroenterology & hepatology 2019;4:445‐453. |  |
| 39. Poupon 1996 | Poupon RE, Huet PM, Poupon R, *et al.* A randomized trial comparing colchicine and ursodeoxycholic acid combination to ursodeoxycholic acid in primary biliary cirrhosis. Hepatology 1996;24(5):1098-1103. | Not a comparator of interest |
| 40. Silveira 2017 | Silveira MG, Gossard AA, Stahler AC, *et al.* A Randomized, Placebo-Controlled Clinical Trial of Efficacy and Safety: Modafinil in the Treatment of Fatigue in Patients With Primary Biliary Cirrhosis. American Journal of Therapeutics 2017;24:e167-e176. | Not a comparator of interest |
| 41. Wolfhagen 1998 | Wolfhagen FH, van Hoogstraten HJ, van Buuren HR, *et al.* Triple therapy with ursodeoxycholic acid, prednisone and azathioprine in primary biliary cirrhosis: a 1-year randomized, placebo-controlled study. Journal of Hepatology 1998;29:736-42. | Not a comparator of interest |

## Summary of IPD analysis of ELATIVE

When the ULN criteria from RESPONSE were applied to ELATIVE, 16 patients were excluded from ELATIVE. The total sample size after adjustment was 145 patients. The baseline characteristics in the intention-to-treat (ITT) population of ELATIVE were compared to the baseline characteristics in the truncated population (Table S 10). No statistically significant differences were observed between the two cohorts; it is worth noting that the mean ALP level at baseline increased (338.0 U/L) in the truncated ELATIVE population compared to the ELATIVE ITT population (321.9 U/L) due to exclusion of patients with the lowest ALP at baseline. The truncated ELATIVE population was deemed comparable to the population in RESPONSE for the NMA of elafibranor versus seladelpar (see Table S 11), negating the need for population adjustment.

The outcomes after aligning the ULN criteria of ELATIVE to RESPONSE are presented in Table S 12.

**Table S 10****: Baseline characteristics in the ELATIVE ITT and the truncated ELATIVE patient populations**

| **Baseline characteristic** | **ELATIVE ITT population (n=161)** | | **Truncated ELATIVE population (n=145)** | | | **P-value*** |
| --- | --- | --- | --- | --- | --- | --- |
|  | **Mean** | **SD** | **Mean** | **95% CI (LCI-UCI)** | **SD** |  |
| Age at diagnosis (years)† | 49.10 | 8.2 | 49.12 | 47.77-50.47 | 8.28 | 0.9831 |
| ALP level (U/L) | 321.9 | 150.9 | 338.0 | 313.53-362.52 | 150.5 | 0.3515 |
| TB (mg/dL) | 0.56 | 0.29 | 0.57 | 0.52-0.62 | 0.37 | 0.7916 |
| Cirrhosis – assessed via liver stiffness (kPa) | 10.14 | 8.16 | 10.32 | 8.94-11.7 | 8.27 | 0.8483 |
| ANA status | NR | NR | NR | NR | NR | NR |

*Calculated using unpaired *t* test. †Calculated from age at baseline and duration of PBC.

Abbreviations: ALP – alkaline phosphatase; ANA – antinuclear antibodies; CI – confidence interval; ITT – intention-to-treat; kPa – kilopascal; LCI – lower confidence interval; mg/dL – milligrams per decilitre; n – number; NR – not reported; SD – standard deviation; TB – total bilirubin; UCI – upper confidence interval; U/L – units per litre

**Table S 11****: Differences in treatment effect modifiers in ELATIVE and RESPONSE**

|  | **ELATIVE†** | | | **RESPONSE** | | |
| --- | --- | --- | --- | --- | --- | --- |
|  | **Mean** | **IQR** | **SD** | **Mean** | **IQR** | **SD** |
| Age at diagnosis (years) | 49.12 | (47.77-50.47) | 8.28 | 49.23 | (47.15-51.31) | 14.72 |
| ALP levels (IU/L) | 338.0 | (313.53-362.52) | 150.5 | 314.3 | (290.31-338.35) | 170.2 |
| Total bilirubin, (mg/dL) | 0.57 | (0.52-0.62) | 0.30 | 0.76 | (0.7-0.82) | 0.42 |
| Cirrhosis assessed via liver stiffness (kPa) | 10.32 | (8.94-11.7) | 8.27 | 9.43 | (8.37-10.49) | 7.49 |
| ANA positive status (%) | NR | NR | NR | NR | NR | NR |

†Truncated ELATIVE population

Abbreviations: ALP – alkaline phosphatase; ANA – antinuclear antibodies; dL – decilitre; IQR – interquartile range; IU – international unit; kPa – kilopascal; L – litre; mg – milligram; n – number; NR – not reported; SD – standard deviation; SMD – standardised mean difference

**Table S 12****: Outcomes in ELATIVE after alignment to RESPONSE eligibility criteria and outcome definitions**

| **Outcome** | **Elafibranor (N=101)** | **Placebo (N=44)** |
| --- | --- | --- |
| Cholestasis response* – n | 58 | 1 |
| Cholestasis response* in patients with ALP ≥350U/L at baseline – n/N | 9/31 | 0/18 |
| ALP normalisation* – n | 17 | 0 |
| LSM CFB in ALP (IU/L) in the ITT population (SE) | -119.881 (7.606) | -0.034 (11.679) |
| All-cause discontinuation – n | 12 | 6 |
| Pruritus as a TEAE – n | 21 | 13 |
| LSM CFB in WI-NRS in the ITT population (SE) | -0.749 (0.167) | -0.362 (0.240) |
| LSM CFB in 5-D Itch in the ITT population (SE) | -1.847 (0.311) | -0.476 (0.483) |
| LSM CFB in PBC-40 Itch in the ITT population (SE) | -1.278 (0.231) | -0.121 (0.356) |
| LSM CFB in WI-NRS in the pruritus ITT population (SE) | -1.766 (0.315) | -0.683 (0.478) |
| LSM CFB in 5-D Itch in the pruritus ITT population (SE) | -4.058 (0.576) | -0.796 (1.007) |
| LSM CFB in PBC-40 Itch in the pruritus ITT population (SE) | -2.241 (0.409) | -0.052 (0.711) |

* ULN defined as 116 U/L and 1.1 mg/dL for ALP and TB, respectively.

Abbreviations: ALP – alkaline phosphatase; CFB – change from baseline; ITT – intention-to-treat; IU – international units; L – litre; LSM – least square mean; n – number; PBC – primary biliary cholangitis; SE – standard error; TB – total bilirubin; ULN – upper limit of normal; WI-NRS – Worst-Itch Numerical Rating Scale

## Summary of data included in the NMA

Table S 13: Summary of data included in the NMA

| **Outcome** | **ELATIVE** | | **RESPONSE** | |
| --- | --- | --- | --- | --- |
|  | **Elafibranor** | **Placebo** | **Seladelpar** | **Placebo** |
| Cholestasis response (ITT population) (n/N) | 58/101 | 1/44 | 79/128 | 13/65 |
| Cholestasis response (ALP ≥ 350 U/L at baseline population) (n/N) | 9/31 | 0/18 | 9/35 | 3/18 |
| ALP normalisation (n/N) | 17/101 | 0/44 | 32/128 | 0/65 |
| Pruritus as a TEAE | 21/101 | 13/44 | 10/128 | 6/65 |
| All-cause discontinuation | 12/101 | 6/44 | 11/128 | 8/65 |
| LSM CFB (SE) in ALP | -119.9 (7.6) | 0.0 (11.7) | -133.9 (8.0)* | -16.9 (11.0)* |
| LSM CFB (SE) in WI-NRS (ITT) | -0.75 (0.17) | -0.36 (0.24) | -1.36 (0.16) | -0.51 (0.43) |
| LSM CFB (SE) in WI-NRS (Pruritus ITT) | -1.77 (0.32) | -0.68 (0.48) | -2.90 (0.58) | -1.29 (0.83) |
| LSM CFB (SE) in 5-D Itch (ITT) | -1.85 (0.31) | -0.48 (0.48) | -2.33 (0.34) | -0.02 (0.90) |
| LSM CFB (SE) in 5-D Itch (Pruritus ITT) | -4.06 (0.58) | -0.80 (1.01) | -5.54 (1.06) | -2.00 (1.50) |
| LSM CFB (SE) in PBC-40 Itch (ITT) | -1.28 (0.23) | -0.12 (0.36) | -1.31 (0.27) | -0.48 (0.71) |
| LSM CFB (SE) in PBC-40 Itch (Pruritus ITT) | -2.24 (0.41) | -0.05 (0.71) | -2.94 (0.94) | -1.42 (1.36) |

*Standard error of change from baseline was derived from the mean baseline ALP value and 95% credible interval of the percentage change from baseline.

Abbreviations: CFB – change from baseline; CrI – credible interval; ITT – intention-to-treat; LSM – least squares mean; OR – odds ratio; PBC – primary biliary cholangitis; SD – standard deviation; TEAE – treatment-emergent adverse event; WI-NRS – worst-itch numerical rating scale

## Further random-effects model results

The median ORs (95% CrI) of pruritus as a TEAE of any severity at 52 weeks for elafibranor-treated patients compared to placebo- or seladelpar-treated patients were 0.63 (0.22, 1.82) and 0.73 (0.14, 3.81), respectively (Figure 6). There were 81.6% and 64.9% posterior probabilities that the odds of pruritus as a TEAE were lower with elafibranor than placebo or seladelpar, respectively (Table 2).

The median ORs (95% CrI) of all-cause discontinuation at 52 weeks for elafibranor-treated patients compared to placebo- or seladelpar-treated patients were 0.88 (0.27, 3.07) and 1.30 (0.25, 6.90), respectively (Figure 7). There were 58.6% and 36.8% posterior probabilities that the odds of all-cause discontinuation of treatment were lower with elafibranor than placebo or seladelpar, respectively (Table 2).

In the ITT population, the median difference in LSM CFB (95% CrI) in pruritus at 52 weeks was -0.39 (-1.17, 0.39), -1.37 (-2.84, 0.08) and -1.16 (-2.25, -0.07) for elafibranor-treated patients compared to placebo-treated patients when measured using a NRS, 5-D Itch and PBC-40 Itch, respectively (Figure 8). There were 85.0%, 96.9% and 98.0% posterior probabilities that pruritus was reduced by a greater extent for elafibranor-treated patients than placebo-treated patients for each of the tools used to measure pruritus, respectively (Table 2). Compared to seladelpar-treated patients, the median difference in LSM CFB (95% CrI) in pruritus was 0.46 (-0.83, 1.75), 0.93 (-1.62, 3.49), and -0.33 (-2.29, 1.64) for elafibranor-treated patients for each of the tools used to measure pruritus, respectively (Figure 8). There were 23.6%, 23.3% and 64.2% posterior probabilities that pruritus was reduced by a greater extent for elafibranor-treated patients than seladelpar-treated patients for each of the tools used to measure pruritus, respectively (Table 2).

In the pruritus ITT population, results were similar to the ITT population. The median difference in LSM CFB (95% CrI) in pruritus at 52 weeks was -1.09 (-2.38, 0.20), -3.26 (-5.79, -0.71), and -2.19 (-3.97, -0.40) for elafibranor-treated patients compared to placebo-treated patients when measured using a NRS, 5-D Itch and PBC-40 Itch, respectively (Figure 8). There were 95.0%, 99.5% and 99.2% posterior probabilities that pruritus was reduced by a greater extent for elafibranor-treated patients than placebo-treated patients for each of the tools used to measure pruritus, respectively (Table 2). Compared to seladelpar-treated patients, the median difference in LSM CFB (95% CrI) in pruritus was 0.52 (-1.92, 2.98), 0.29 (-4.22, 4.81) and -0.67 (-4.43, 3.11) for elafibranor-treated patients compared to seladelpar-treated patients for each of the tools used to measure pruritus, respectively (Figure 8). There were 33.3%, 44.8%, and 64.0% posterior probabilities that pruritus was reduced by a greater extent for elafibranor-treated patients than seladelpar-treated patients for each of the tools used to measure pruritus, respectively (Table 2).

## Treatment effect matrices

Table S 14: Treatment effect matrices for all outcomes – random-effects base case models

| **Outcome** | **Reference treatment (denominator)** | **Comparative treatment (numerator)** | | |
| --- | --- | --- | --- | --- |
|  |  | Elafibranor | Seladelpar | Placebo |
| Median OR (95% CrI) of cholestasis response (ITT population) | Elafibranor | 1 | 0.08 (0.00, 0.69) | 0.01 (0.00, 0.08) |
|  | Seladelpar | 13.02 (1.45, 420.20) | 1 | 0.15 (0.06, 0.40) |
|  | Placebo | 84.79 (12.49, 2,513.00) | 6.63 (2.52, 18.14) | 1 |
| Median OR (95% CrI) of cholestasis response (ALP ≥ 350 U/L at baseline population) | Elafibranor | 1 | 0.05 (0.00, 1.54) | 0.03 (0.00, 0.46) |
|  | Seladelpar | 18.71 (0.65, 10,610.00) | 1 | 0.54 (0.09, 2.59) |
|  | Placebo | 33.52 (2.17, 17,040.00) | 1.87 (0.39, 11.35) | 1 |
| Median OR (95% CrI) of ALP normalisation | Elafibranor | 1 | 2.38 (0.00, 1,558.03) | 0.02 (0.00, 0.35) |
|  | Seladelpar | 0.42 (0.00, 282.30) | 1 | 0.01 (0.00, 0.14) |
|  | Placebo | 40.54 (2.89, 18,880.25) | 96.11 (7.35, 40,250.50) | 1 |
| Median difference in LSM CFB (95% CrI) in ALP | Elafibranor | 0 | -1.87 (-53.77, 48.59) | 115.20 (77.34, 151.10) |
|  | Seladelpar | 1.87 (-48.59, 53.77) | 0 | 117.10 (79.97, 154.20) |
|  | Placebo | -115.20 (-151.10, -77.34) | -117.10 (-154.20, -79.97) | 0 |
| Median OR of pruritus as a TEAE (95% CrI) | Elafibranor | 1 | 1.37 (0.26, 7.38) | 1.60 (0.55, 4.55) |
|  | Seladelpar | 0.73 (0.14, 3.81) | 1 | 1.17 (0.31, 4.11) |
|  | Placebo | 0.63 (0.22, 1.82) | 0.86 (0.24, 3.20) | 1 |
| Median OR of all-cause discontinuation (95% CrI) | Elafibranor | 1 | 0.77 (0.14, 3.95) | 1.14 (0.33, 3.69) |
|  | Seladelpar | 1.30 (0.25, 6.90) | 1 | 1.48 (0.47, 4.47) |
|  | Placebo | 0.88 (0.27, 3.07) | 0.67 (0.22, 2.11) | 1 |
| Median difference in LSM CFB (95% CrI) in a pruritus NRS (ITT) | Elafibranor | 0 | -0.46 (-1.75, 0.83) | 0.39 (-0.39, 1.17) |
|  | Seladelpar | 0.46 (-0.83, 1.75) | 0 | 0.85 (-0.19, 1.89) |
|  | Placebo | -0.39 (-1.17, 0.39) | -0.85 (-1.89, 0.19) | 0 |
| Median difference in LSM CFB (95% CrI) in a pruritus NRS (Pruritus ITT) | Elafibranor | 0 | -0.52 (-2.98, 1.92) | 1.09 (-0.20, 2.38) |
|  | Seladelpar | 0.52 (-1.92, 2.98) | 0 | 1.61 (-0.47, 3.70) |
|  | Placebo | -1.09 (-2.38, 0.20) | -1.61 (-3.70, 0.47) | 0 |
| Median difference in LSM CFB (95% CrI) in 5-D Itch (ITT) | Elafibranor | 0 | -0.93 (-3.49, 1.62) | 1.37 (-0.08, 2.84) |
|  | Seladelpar | 0.93 (-1.62, 3.49) | 0 | 2.30 (0.22, 4.41) |
|  | Placebo | -1.37 (-2.84, 0.08) | -2.30 (-4.41, -0.22) | 0 |
| Median difference in LSM CFB (95% CrI) in 5-D Itch (Pruritus ITT) | Elafibranor | 0 | -0.29 (-4.81, 4.22) | 3.26 (0.71, 5.79) |
|  | Seladelpar | 0.29 (-4.22, 4.81) | 0 | 3.54 (-0.21, 7.31) |
|  | Placebo | -3.26 (-5.79, -0.71) | -3.54 (-7.31, 0.21) | 0 |
| Median difference in LSM CFB (95% CrI) in PBC-40 Itch (ITT) | Elafibranor | 0 | 0.33 (-1.64, 2.29) | 1.16 (0.07, 2.25) |
|  | Seladelpar | -0.33 (-2.29, 1.64) | 0 | 0.83 (-0.81, 2.47) |
|  | Placebo | -1.16 (-2.25, -0.07) | -0.83 (-2.47 0.81) | 0 |
| Median difference in LSM CFB (95% CrI) in PBC-40 Itch (Pruritus ITT) | Elafibranor | 0 | 0.67 (-3.11, 4.43) | 2.19 (0.04, 3.97) |
|  | Seladelpar | -0.67 (-4.43, 3.11) | 0 | 1.52 (-1.80, 4.85) |
|  | Placebo | -2.19 (-3.97, -0.40) | -1.52 (-4.85, 1.80) | 0 |

Abbreviations: CFB – change from baseline; CrI – credible interval; ITT – intention-to-treat; LSM – least squares mean; NRS – numerical rating scale; OR – odds ratio; PBC – primary biliary cholangitis; SD – standard deviation; TEAE – treatment-emergent adverse event

Table S 15: Treatment effect matrices for all outcomes – fixed-effect models

| **Outcome** | **Reference treatment (denominator)** | **Comparative treatment (numerator)** | | |
| --- | --- | --- | --- | --- |
|  |  | Elafibranor | Seladelpar | Placebo |
| Median OR (95% CrI) of cholestasis response (ITT population) | Elafibranor | 1 | 0.08 (0.00, 0.55) | 0.01 (0.00, 0.07) |
|  | Seladelpar | 12.77 (1.83, 390.70) | 1 | 0.15 (0.07, 0.30) |
|  | Placebo | 83.98 (14.26, 2433.00) | 6.61 (3.34, 13.96) | 1 |
| Median OR (95% CrI) of cholestasis response (ALP ≥ 350 U/L at baseline population) | Elafibranor | 1 | 0.05 (0.00, 1.32) | 0.03 (0.00, 0.42) |
|  | Seladelpar | 18.46 (0.76, 9,494.02) | 1 | 0.54 (0.10, 2.23) |
|  | Placebo | 33.13 (2.39, 15,750.00) | 1.86 (0.45, 9.95) | 1 |
| Median OR (95% CrI) of ALP normalisation | Elafibranor | 1 | 2.44 (0.00, 1,620.00) | 0.03 (0.00, 0.31) |
|  | Seladelpar | 0.41 (0.00, 268.20) | 1 | 0.01 (0.00, 0.12) |
|  | Placebo | 39.01 (3.22, 18,130.00) | 95.68 (8.14, 42,410.00) | 1 |
| Median OR of pruritus as a TEAE (95% CrI) | Elafibranor | 1 | 1.36 (0.35, 5.50) | 1.60 (0.70, 3.59) |
|  | Seladelpar | 0.74 (0.18, 2.83) | 1 | 1.18 (0.38, 3.38) |
|  | Placebo | 0.63 (0.28, 1.43) | 0.85 (0.30, 2.66) | 1 |
| Median OR of all-cause discontinuation (95% CrI) | Elafibranor | 1 | 0.77 (0.17, 3.29) | 1.14 (0.37, 3.22) |
|  | Seladelpar | 1.30 (0.30, 5.74) | 1 | 1.48 (0.54, 3.92) |
|  | Placebo | 0.87 (0.31, 2.72) | 0.68 (0.26, 1.86) | 1 |
| Median difference in LSM CFB (95% CrI) in ALP | Elafibranor | 0 | -0.36 (-37.39, 36.89) | 116.90 (90.11, 143.60) |
|  | Seladelpar | 0.36 (-36.89, 37.39) | 0 | 117.20 (90.80, 143.40) |
|  | Placebo | -116.90 (-143.60, -90.11) | -117.20 (-143.40, -90.80) | 0 |
| Median difference in LSM CFB (95% CrI) in a pruritus NRS (ITT) | Elafibranor | 0 | -0.46 (-1.52, 0.61) | 0.39 (-0.19, 0.97) |
|  | Seladelpar | 0.46 (-0.61, 1.52) | 0 | 0.85 (-0.05, 1.75) |
|  | Placebo | -0.39 (-0.97, 0.19) | -0.85 (-1.75, 0.05) | 0 |
| Median difference in LSM CFB (95% CrI) in a pruritus NRS (Pruritus ITT) | Elafibranor | 0 | -0.52 (-2.80, 1.77) | 1.09 (-0.04, 2.22) |
|  | Seladelpar | 0.52 (-1.77, 2.80) | 0 | 1.61 (-0.38, 3.59) |
|  | Placebo | -1.09 (-2.22, 0.04) | -1.61 (-3.59, 0.38) | 0 |
| Median difference in LSM CFB (95% CrI) in 5-D Itch (ITT) | Elafibranor | 0 | -0.94 (-3.12, 1.25) | 1.37 (0.25, 2.49) |
|  | Seladelpar | 0.94 (-1.25, 3.12) | 0 | 2.31 (0.42, 4.18) |
|  | Placebo | -1.37 (-2.49, -0.25) | -2.31 (-4.18, -0.42) | 0 |
| Median difference in LSM CFB (95% CrI) in 5-D Itch (Pruritus ITT) | Elafibranor | 0 | -0.29 (-4.53, 4.00) | 3.26 (0.98, 5.54) |
|  | Seladelpar | 0.29 (-4.00, 4.53) | 0 | 3.55 (-0.04, 7.14) |
|  | Placebo | -3.26 (-5.54, -0.98) | -3.55 (-7.14, 0.04) | 0 |
| Median difference in LSM CFB (95% CrI) in PBC-40 Itch (ITT) | Elafibranor | 0 | 0.33 (-1.38, 2.04) | 1.16 (0.33, 2.00) |
|  | Seladelpar | -0.33 (-2.04, 1.38) | 0 | 0.83 (-0.66, 2.31) |
|  | Placebo | -1.16 (-2.00, -0.33) | -0.83 (-2.31, 0.66) | 0 |
| Median difference in LSM CFB (95% CrI) in PBC-40 Itch (Pruritus ITT) | Elafibranor | 0 | 0.67 (-2.94, 4.28) | 2.19 (0.58, 3.80) |
|  | Seladelpar | -0.67 (-4.28, 2.94) | 0 | 1.52 (-1.73, 4.76) |
|  | Placebo | -2.19 (-3.80, -0.58) | -1.52 (-4.76, 1.73) | 0 |

Abbreviations: CFB – change from baseline; CrI – credible interval; ITT – intention-to-treat; LSM – least squares mean; NRS –numerical rating scale; OR – odds ratio; PBC – primary biliary cholangitis; SD – standard deviation; TEAE – treatment-emergent adverse event

## Random-effects model settings

The model settings for the random-effects model NMA results are presented below in Table S 16**Error! Reference source not found.**.

Table S 16: Model settings for random-effects model NMAs

| **Outcome** | **Population** | **Burn-in** | **Number of iterations** | **Thinning interval** |
| --- | --- | --- | --- | --- |
| Cholestasis response | ITT | 250,000 | 450,000 | 20 |
|  | ALP ≥ 350 U/L | 50,000 | 300,000 | 25 |
| ALP normalisation | ITT | 150,000 | 300,000 | 20 |
| CFB in ALP | ITT | 100,000 | 200,000 | 10 |
| Pruritus as TEAE | ITT | 50,000 | 200,000 | 7 |
| All-cause discontinuation | ITT | 150,000 | 350,000 | 10 |
| Pruritus NRS | ITT | 100,000 | 300,000 | 10 |
|  | Pruritus ITT | 100,000 | 300,000 | 10 |
| 5-D Itch | ITT | 100,000 | 150,000 | 15 |
|  | Pruritus ITT | 150,000 | 200,000 | 10 |
| PBC-40 Itch | ITT | 100,000 | 200,000 | 15 |
|  | Pruritus ITT | 100,000 | 250,000 | 10 |

Abbreviations: ALP – alkaline phosphatase; CFB – change from baseline; ITT – intention-to-treat; LSM – least-square mean; NMA – network meta-analysis; NRS – numerical rating scale; PBC – primary biliary cholangitis; TEAE – treatment-emergent adverse event; U/L – units per litre

## Fixed-effect model results

As a sensitivity analysis to the random-effects model run in the base-case, a fixed-effect model was run, assuming that treatment effects are identical across studies, ignoring potential heterogeneity.^13^ Model settings for all fixed-effects analyses for pruritus outcomes are presented in Table S 17.

Under the fixed-effect analysis, there were greater odds of achieving cholestasis response at 52 weeks in the ITT population for elafibranor-treated patients compared to placebo- (median OR [95% CrI]: 83.98 [14.26, 2,433.00]) or seladelpar-treated patients (12.77 [1.83, 390.70]; Figure S 1). The posterior probabilities that the odds were higher with elafibranor than placebo or seladelpar were 100.0% and 99.7%, respectively (Table S 18).

Consistent with the ITT population, there were greater odds of achieving cholestasis response at 52 weeks in the ALP≥350 U/L sub-population for elafibranor-treated patients than placebo- (33.13 [2.39, 15,7500.00]) or seladelpar-treated patients (18.46 [0.76, 9,494.02]; Figure S 1). There were 99.8% and 96.0% posterior probabilities that the odds were higher with elafibranor than placebo or seladelpar, respectively (Table S 18).

The median ORs (95% CrI) of ALP normalisation at 52 weeks for elafibranor-treated patients compared to placebo- or seladelpar-treated patients were 39.01 (3.22, 18,130.00) and 0.41 (0.00, 286.20), respectively (Figure S 2). There were 100.0% and 35.2% posterior probabilities that the odds were higher with elafibranor than placebo or seladelpar, respectively (Table S 18). Likewise, the median difference in LSM CFB (95% CrI) in ALP at 52 weeks for elafibranor-treated patients compared to placebo- or seladelpar-treated patients were -116.90 (-143.60, -90.11) and 0.36 (-36.89, 37.39), respectively (Figure S 3). There were 100.0% and 48.9% posterior probabilities that the reduction was greater for elafibranor than placebo or seladelpar, respectively (Table S 18).

The median ORs (95% CrI) of pruritus as a TEAE at 52 weeks for elafibranor-treated patients compared to placebo- or seladelpar-treated patients were 0.63 (0.28, 1.43) and 0.74 (0.18, 2.83), respectively (Figure S 4). There were 87.2% and 67.2% posterior probabilities that the odds of pruritus as a TEAE were lower with elafibranor than placebo or seladelpar, respectively (Table S 18).

The median OR (95% CrI) of all-cause discontinuation for elafibranor-treated patients compared to placebo- or seladelpar-treated patients were 0.87 (0.31, 2.72), and 1.30 (0.30, 5.74), respectively (Figure S 5). There were 60.4% and 36.7% posterior probabilities that the odds of all-cause discontinuation of treatment were lower with elafibranor than placebo or seladelpar, respectively (Table S 18).

In the ITT population, the median difference in LSM CFB (95% CrI) in pruritus at 52 weeks was -0.39 (-0.97, 0.19), -1.37 (-2.49, -0.25), and -1.16 (-2.00, -0.33) for elafibranor-treated patients compared to placebo-treated patients when measured using a NRS, 5-D Itch and PBC-40 Itch, respectively (Figure S 6). There were 90.8%, 99.3% and 99.6% posterior probabilities that pruritus was reduced by a greater extent for elafibranor-treated patients than placebo-treated patients for each of the tools used to measure pruritus, respectively (Table S 18). Compared to seladelpar-treated patients, the median difference in LSM CFB (95% CrI) in pruritus was 0.46 (-0.61, 1.52), 0.94 (-1.25, 3.12), and -0.33 (-2.04, 1.38) for elafibranor-treated patients for each of the tools used to measure pruritus, respectively (Figure S 6). There were 19.9%, 19.9% and 65.2% posterior probabilities that pruritus was reduced by a greater extent for elafibranor-treated patients than seladelpar-treated patients for each of the tools used to measure pruritus, respectively (Table S 18).

In the pruritus ITT population, results were similar to the ITT population. The median difference in LSM CFB (95% CrI) in pruritus at 52 weeks was -1.09 (-2.22, 0.04), -3.26 (-5.54, -0.98) and -2.19 (-3.80, -0.58) for elafibranor-treated patients compared to placebo-treated patients when measured using a NRS, 5-D Itch and PBC-40 Itch, respectively (Figure S 6). There were 97.1%, 99.8% and 99.6% posterior probabilities that pruritus was reduced by a greater extent for elafibranor-treated patients than placebo-treated patients, respectively (Table S 18). Compared to seladelpar-treated patients, the median difference in LSM CFB (95% CrI) in pruritus was 0.52 (-1.77, 2.80), 0.29 (‑4.00, 4.53) and -0.67 (-4.28, 2.94) for elafibranor-treated patients for each of the tools used to measure pruritus, respectively (Figure S 6). There were 32.5%, 44.5% and 64.0% posterior probabilities that pruritus was reduced by a greater extent for elafibranor-treated patients than seladelpar-treated patients for each of the tools used to measure pruritus, respectively (Table S 18).

**Figure S 1****: Odds ratio of achieving cholestasis response at 52 weeks (fixed effects model)**


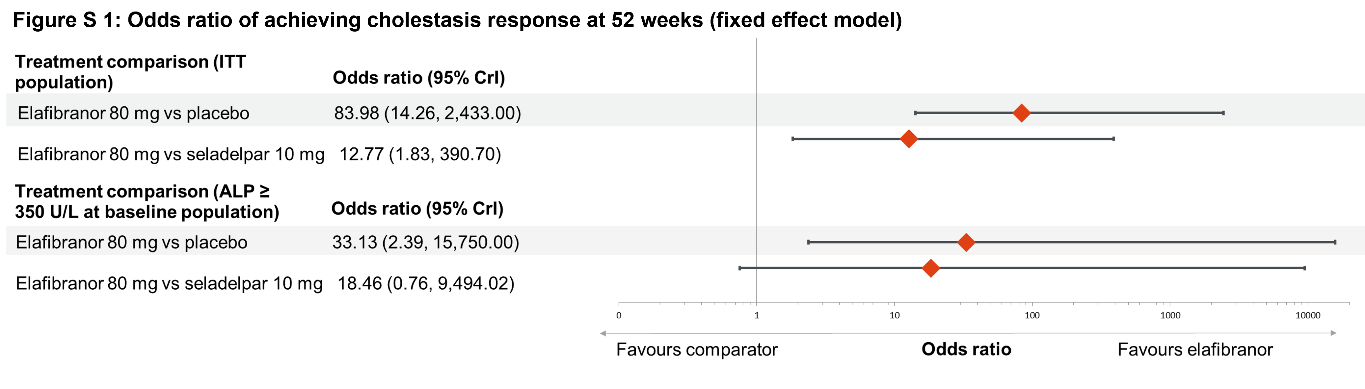


Abbreviations: ALP – alkaline phosphatase; CrI – credible interval; ITT – intention-to-treat; mg – milligram; U/L – units per litre

**Figure S 2****: Odds ratio of achieving ALP normalisation at 52 weeks in the ITT population (fixed effects model)**


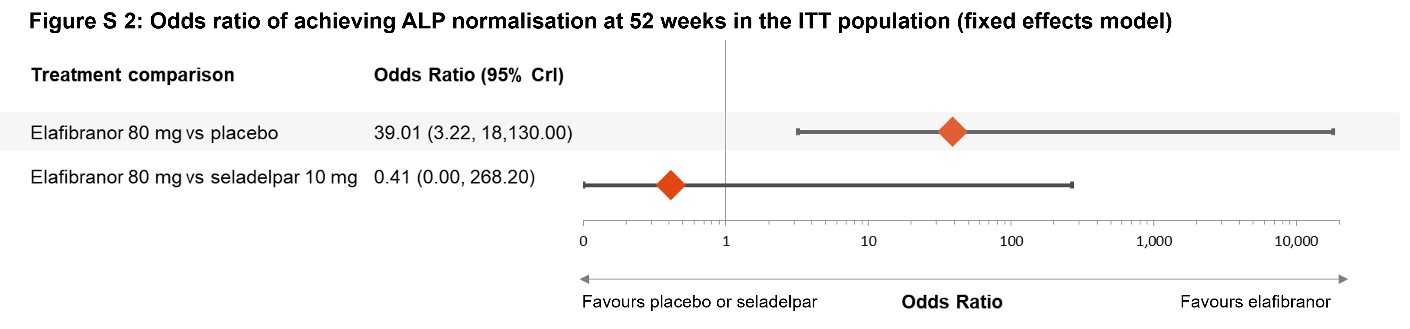


Abbreviations: ALP – alkaline phosphatase; CrI – credible interval; ITT – intention-to-treat; mg – milligram

**Figure S 3****: Median difference in LSM CFB in ALP at 52 weeks in the ITT population (fixed effect model)**


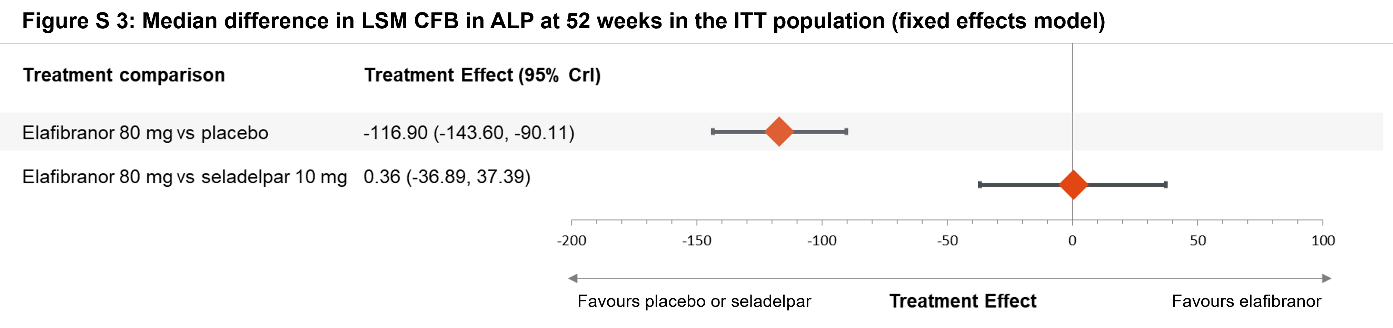


Abbreviations: ALP – alkaline phosphatase; CrI – credible interval; ITT – intention-to-treat; LSM – least-square mean; mg – milligram

**Figure S 4****: Odds ratio of occurrence of pruritus as a TEAE at 52 weeks in the ITT population (fixed effect model)**


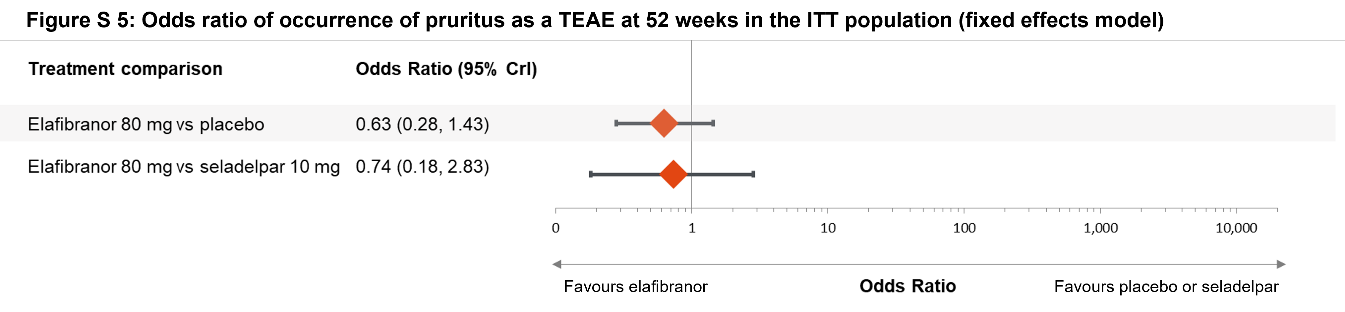


Abbreviations: CrI – credible interval; ITT – intention-to-treat; mg – milligram; TEAE – treatment-emergent adverse event

**Figure S 5****: Odds ratio of all-cause discontinuation at 52 weeks in the ITT population (fixed effect model)**


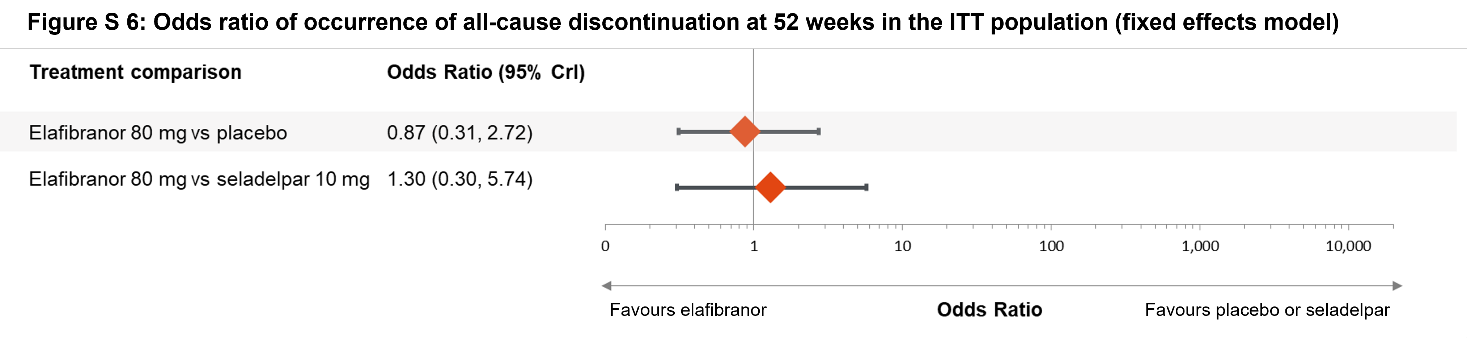
 Abbreviations: CrI – credible interval; ITT – intention-to-treat; mg – milligram

**Figure S 6****: Median difference in LSM CFB for pruritus outcomes at 52 weeks (fixed effect models)**


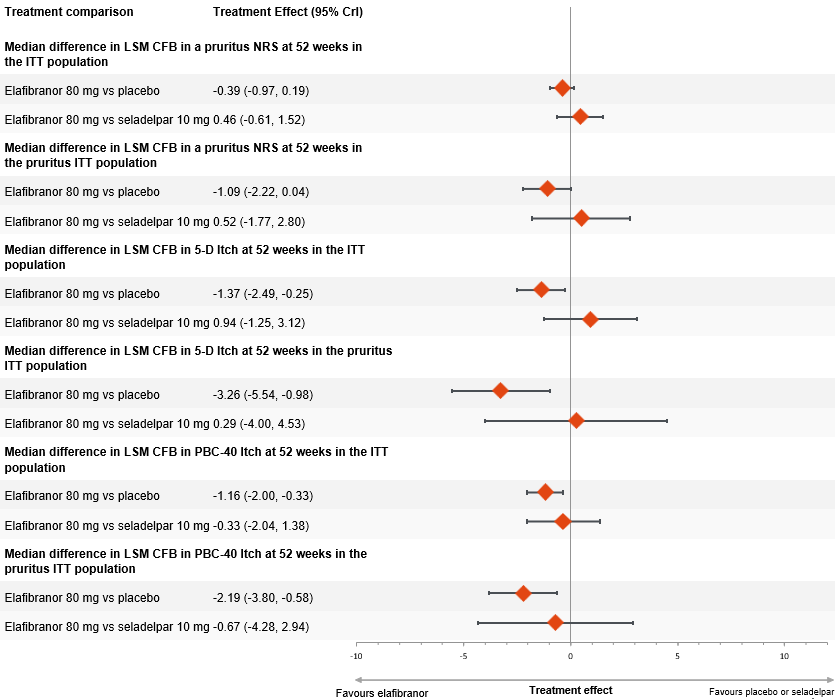


Abbreviations: CFB – change from baseline; CrI – credible interval; ITT – intention-to-treat; LSM – least-square mean; mg – milligram; NRS – numerical rating scale; PBC – primary biliary cholangitis

**Table S 17****: Model settings for random effect NMAs**

| **Outcome** | **Population** | **Burn-in** | **Number of iterations** | **Thinning interval** |
| --- | --- | --- | --- | --- |
| Cholestasis response | ITT | 250,000 | 450,000 | 20 |
|  | ALP ≥ 350 U/L | 50,000 | 300,000 | 25 |
| ALP normalisation | ITT | 150,000 | 300,000 | 20 |
| CFB in ALP | ITT | 100,000 | 200,000 | 10 |
| Pruritus as TEAE | ITT | 50,000 | 200,000 | 7 |
| All-cause discontinuation | ITT | 150,000 | 350,000 | 10 |
| A pruritus NRS | ITT | 100,000 | 300,000 | 10 |
|  | Pruritus ITT | 100,000 | 300,000 | 10 |
| 5-D Itch | ITT | 100,000 | 150,000 | 15 |
|  | Pruritus ITT | 150,000 | 200,000 | 10 |
| PBC-40 Itch | ITT | 100,000 | 200,000 | 15 |
|  | Pruritus ITT | 50,000 | 250,000 | 10 |

Abbreviations: ALP – alkaline phosphatase; CFB – change from baseline; ITT – intention-to-treat; LSM – least-square mean; NMA – network meta-analysis; NRS – numerical rating scale; PBC – primary biliary cholangitis; TEAE – treatment-emergent adverse event; U/L – units per litre

**Table S 18****: Summary statistics from the fixed effect NMA**

| **Analysis** | **Total residual deviance†** | **Posterior probability of elafibranor being preferred to placebo** | **Posterior probability of elafibranor being preferred to seladelpar** |
| --- | --- | --- | --- |
| Cholestasis response (ITT population) | 3.519 | 1.000 | 0.997 |
| Cholestasis response (ALP ≥ 350 U/L at baseline population) | 4.369 | 0.998 | 0.960 |
| ALP normalisation | 3.851 | 1.000 | 0.352 |
| CFB in ALP | 3.342 | 1.000 | 0.489 |
| Pruritus as a TEAE | 4.064 | 0.872 | 0.672 |
| All-cause discontinuation | 3.420 | 0.604 | 0.367 |
| CFB in a pruritus NRS (ITT) | 3.359 | 0.908 | 0.199 |
| CFB in a pruritus NRS (Pruritus ITT) | 3.359 | 0.971 | 0.325 |
| CFB in 5-D Itch (ITT) | 3.360 | 0.993 | 0.199 |
| CFB in 5-D Itch (Pruritus ITT) | 4.012 | 0.998 | 0.445 |
| CFB in PBC-40 Itch (ITT) | 3.354 | 0.996 | 0.652 |
| CFB in PBC-40 Itch (Pruritus ITT) | 3.361 | 0.996 | 0.640 |

†Between-study SD on mean difference scale was used for continuous outcomes, while the OR scale was used for binary outcomes

Abbreviations: CFB – change from baseline; ITT – intention-to-treat; NRS – numerical rating scale; OR – odds ratio; PBC – primary biliary cholangitis; SD – standard deviation; TEAE – treatment-emergent adverse event

## Ongoing studies of elafibranor and seladelpar

Table S 19: Ongoing studies of elafibranor and seladelpar

| **Intervention** | **NCT number** | **Study name** |
| --- | --- | --- |
| Elafibranor | NCT06730061 | ELONSEN |
|  | NCT06383403 | ELSPIRE |
|  | NCT06016842 | ELFIDENCE |
|  | NCT06447168 | ELFINITY |
| Seladelpar | NCT06051617 | AFFIRM |
|  | NCT06060665 | IDEAL |
|  | NCT03301506 | ASSURE |

## PRISMA checklist

The PRISMA checklist for NMAs is presented in Table S 20.

Table S 20: PRISMA checklist for NMAs

| **Section/Topic** | **Item #** | **Checklist Item** | **Reported on Page #** |
| --- | --- | --- | --- |
| **TITLE** |  |  |  |
| Title | 1 | Identify the report as a systematic review *incorporating a network meta-analysis (or related form of meta-analysis).* | 2 |
|  |  |  |  |
| **ABSTRACT** |  |  |  |
| Structured summary | 2 | Provide a structured summary including, as applicable:  **Background:** main objectives  **Methods:** data sources; study eligibility criteria, participants, and interventions; study appraisal; and *synthesis methods, such as network meta-analysis.*  **Results:** number of studies and participants identified; summary estimates with corresponding confidence/credible intervals; *treatment rankings may also be discussed. Authors may choose to summarize pairwise comparisons against a chosen treatment included in their analyses for brevity.*  **Discussion/Conclusions:** limitations; conclusions and implications of findings.  **Other:** primary source of funding; systematic review registration number with registry name. | 4-5 |
|  |  |  |  |
| **INTRODUCTION** |  |  |  |
| Rationale | 3 | Describe the rationale for the review in the context of what is already known*, including mention of why a network meta-analysis has been conducted.* | 7-8 |
| Objectives | 4 | Provide an explicit statement of questions being addressed, with reference to participants, interventions, comparisons, outcomes, and study design (PICOS). | 8 |
|  |  |  |  |
| **METHODS** |  |  |  |
| Protocol and registration | 5 | Indicate whether a review protocol exists and if and where it can be accessed (e.g., Web address); and, if available, provide registration information, including registration number. | 9 |
| Eligibility criteria | 6 | Specify study characteristics (e.g., PICOS, length of follow-up) and report characteristics (e.g., years considered, language, publication status) used as criteria for eligibility, giving rationale. *Clearly describe eligible treatments included in the treatment network, and note whether any have been clustered or merged into the same node (with justification).* | 9-10 |
| Information sources | 7 | Describe all information sources (e.g., databases with dates of coverage, contact with study authors to identify additional studies) in the search and date last searched. | 9-10 |
| Search | 8 | Present full electronic search strategy for at least one database, including any limits used, such that it could be repeated. | Supplement: 5-12 |
| Study selection | 9 | State the process for selecting studies (i.e., screening, eligibility, included in systematic review, and, if applicable, included in the meta-analysis). | 9-10 |
| Data collection process | 10 | Describe method of data extraction from reports (e.g., piloted forms, independently, in duplicate) and any processes for obtaining and confirming data from investigators. | 10 |
| Data items | 11 | List and define all variables for which data were sought (e.g., PICOS, funding sources) and any assumptions and simplifications made. | Supplement: 3-4 |
| **Geometry of the network** | **S1** | Describe methods used to explore the geometry of the treatment network under study and potential biases related to it. This should include how the evidence base has been graphically summarized for presentation, and what characteristics were compiled and used to describe the evidence base to readers. | 10 |
| Risk of bias within individual studies | 12 | Describe methods used for assessing risk of bias of individual studies (including specification of whether this was done at the study or outcome level), and how this information is to be used in any data synthesis. | 10 |
| Summary measures | 13 | State the principal summary measures (e.g., risk ratio, difference in means). *Also describe the use of additional summary measures assessed, such as treatment rankings and surface under the cumulative ranking curve (SUCRA) values, as well as modified approaches used to present summary findings from meta-analyses.* | 12 |
| Planned methods of analysis | 14 | Describe the methods of handling data and combining results of studies for each network meta-analysis. This should include, but not be limited to:   - *Handling of multi-arm trials;* - *Selection of variance structure;* - *Selection of prior distributions in Bayesian analyses; and* - *Assessment of model fit.* | 11-13, supplement: 14-16 |
| **Assessment of Inconsistency** | **S2** | Describe the statistical methods used to evaluate the agreement of direct and indirect evidence in the treatment network(s) studied. Describe efforts taken to address its presence when found. | 11 |
| Risk of bias across studies | 15 | Specify any assessment of risk of bias that may affect the cumulative evidence (e.g., publication bias, selective reporting within studies). | 10 |
| Additional analyses | 16 | Describe methods of additional analyses if done, indicating which were pre-specified. This may include, but not be limited to, the following:   - Sensitivity or subgroup analyses; - Meta-regression analyses; - *Alternative formulations of the treatment network; and* - *Use of alternative prior distributions for Bayesian analyses (if applicable).* | 12 |
|  |  |  |  |
| **RESULTS** |  |  |  |
| Study selection | 17 | Give numbers of studies screened, assessed for eligibility, and included in the review, with reasons for exclusions at each stage, ideally with a flow diagram. | 13 |
| **Presentation of network structure** | **S3** | Provide a network graph of the included studies to enable visualization of the geometry of the treatment network. | 13 |
| **Summary of network geometry** | **S4** | Provide a brief overview of characteristics of the treatment network. This may include commentary on the abundance of trials and randomized patients for the different interventions and pairwise comparisons in the network, gaps of evidence in the treatment network, and potential biases reflected by the network structure. | 13 |
| Study characteristics | 18 | For each study, present characteristics for which data were extracted (e.g., study size, PICOS, follow-up period) and provide the citations. | Table 1, 23 |
| Risk of bias within studies | 19 | Present data on risk of bias of each study and, if available, any outcome level assessment. | Table 1, 24-25 |
| Results of individual studies | 20 | For all outcomes considered (benefits or harms), present, for each study: 1) simple summary data for each intervention group, and 2) effect estimates and confidence intervals. *Modified approaches may be needed to deal with information from larger networks.* | Supplement: 20-21 |
| Synthesis of results | 21 | Present results of each meta-analysis done, including confidence/credible intervals. *In larger networks, authors may focus on comparisons versus a particular comparator (e.g. placebo or standard care), with full findings presented in an appendix. League tables and forest plots may be considered to summarize pairwise comparisons.* If additional summary measures were explored (such as treatment rankings), these should also be presented. | 14-15 |
| **Exploration for inconsistency** | **S5** | Describe results from investigations of inconsistency. This may include such information as measures of model fit to compare consistency and inconsistency models, *P* values from statistical tests, or summary of inconsistency estimates from different parts of the treatment network. | 15, supplement: 32-33 |
| Risk of bias across studies | 22 | Present results of any assessment of risk of bias across studies for the evidence base being studied. | Table 1, 24-25 |
| Results of additional analyses | 23 | Give results of additional analyses, if done (e.g., sensitivity or subgroup analyses, meta-regression analyses*, alternative network geometries studied, alternative choice of prior distributions for Bayesian analyses,* and so forth). | Supplement: 20-21, 27-32 |
|  |  |  |  |
| **DISCUSSION** |  |  |  |
| Summary of evidence | 24 | Summarize the main findings, including the strength of evidence for each main outcome; consider their relevance to key groups (e.g., healthcare providers, users, and policy-makers). | 16 |
| Limitations | 25 | Discuss limitations at study and outcome level (e.g., risk of bias), and at review level (e.g., incomplete retrieval of identified research, reporting bias). *Comment on the validity of the assumptions, such as transitivity and consistency. Comment on any concerns regarding network geometry (e.g., avoidance of certain comparisons).* | 16-18 |
| Conclusions | 26 | Provide a general interpretation of the results in the context of other evidence, and implications for future research. | 19 |
|  |  |  |  |
| **FUNDING** |  |  |  |
| Funding | 27 | Describe sources of funding for the systematic review and other support (e.g., supply of data); role of funders for the systematic review. This should also include information regarding whether funding has been received from manufacturers of treatments in the network and/or whether some of the authors are content experts with professional conflicts of interest that could affect use of treatments in the network. | 2 |

PICOS = population, intervention, comparators, outcomes, study design.

## References

1. Study of Elafibranor in Patients With Primary Biliary Cholangitis (PBC) - Full Text View - ClinicalTrials.gov. at <https://clinicaltrials.gov/ct2/show/NCT04526665>

2. CymaBay Therapeutics, Inc. *RESPONSE: A Placebo-controlled, Randomized, Phase 3 Study to Evaluate the Efficacy and Safety of Seladelpar in Patients With Primary Biliary Cholangitis (PBC) and an Inadequate Response to or an Intolerance to Ursodeoxycholic Acid (UDCA)*. (clinicaltrials.gov, 2023). at <https://clinicaltrials.gov/study/NCT04620733>

3. Kowdley KV, Bowlus CL, Levy C, *et al.* Efficacy and Safety of Elafibranor in Primary Biliary Cholangitis. *N Engl J Med* 2023. doi:10.1056/NEJMoa2306185

4. Hirschfield GM, Bowlus CL, Mayo MJ, *et al.* A Phase 3 Trial of Seladelpar in Primary Biliary Cholangitis. *N Engl J Med* 2024. 390: 783–794.

5. Markov Chain Monte Carlo. *Columbia University Mailman School of Public Health* 2016. at <https://www.publichealth.columbia.edu/research/population-health-methods/markov-chain-monte-carlo>

6. Rhodes KM, Turner RM, White IR, *et al.* Implementing informative priors for heterogeneity in meta‐analysis using meta‐regression and pseudo data. *Stat Med* 2016. 35: 5495–5511.

7. Ren S, Oakley JE & Stevens JW. Incorporating Genuine Prior Information about Between-Study Heterogeneity in Random Effects Pairwise and Network Meta-analyses. *Med Decis Making* 2018. 38: 531–542.

8. Brooks SP & Gelman A. General Methods for Monitoring Convergence of Iterative Simulations. *Journal of Computational and Graphical Statistics* 1998. 7: 434–455.

9. Bayesian analysis: Gelman–Rubin convergence diagnostic | Stata. at <https://www.stata.com/features/overview/gelman-rubin-convergence-diagnostic/>

10. Burn-in Period - an overview | ScienceDirect Topics. at <https://www.sciencedirect.com/topics/mathematics/burn-in-period>

11. NICE TSU. NICE TSU: Frequently asked questions (FAQs) regarding evidence synthesis. at <https://www.bristol.ac.uk/media-library/sites/social-community-medicine/documents/mpes/TSU%20Frequently%20asked%20questions_Dec2019.pdf>

12. Barili F, Parolari A, Kappetein PA, *et al.* Statistical Primer: heterogeneity, random- or fixed-effects model analyses?†. *Interactive CardioVascular and Thoracic Surgery* 2018. 27: 317–321.

13. Dettori JR, Norvell DC & Chapman JR. Fixed-Effect vs Random-Effects Models for Meta-Analysis: 3 Points to Consider. *Global Spine J* 2022. 12: 1624–1626.

14. Bowlus CL, Choi Y-J, Yang K, *et al.* Seladelpar improved the lipid profile of patients with primary biliary cholangitis (pbc): results from phase 2 and 3 clinical studies. *In Hepatology, WILEY 111 River St, Hoboken 07030-5774, NJ USA* 2022. at <https://content.equisolve.net/cymabay/media/c0307e5a76cb044228c7ac7ae788f382.pdf>

15. Hirschfield GM, Shiffman ML, Gulamhusein A, *et al.* Seladelpar efficacy and safety at 3 months in patients with primary biliary cholangitis: ENHANCE, a phase 3, randomized, placebo-controlled study. *Hepatology* 2023. 78: 397–415.

16. Bowlus CL, Galambos MR, Aspinall RJ, *et al.* A phase II, randomized, open-label, 52-week study of seladelpar in patients with primary biliary cholangitis. *J Hepatol* 2022. 77: 353–364.

17. Schattenberg JM, Pares A, Kowdley KV, *et al.* A randomized placebo-controlled trial of elafibranor in patients with primary biliary cholangitis and incomplete response to UDCA. *J Hepatol* 2021. 74: 1344–1354.

18. Jones D, Boudes PF, Swain MG, *et al.* Seladelpar (MBX-8025), a selective PPAR-δ agonist, in patients with primary biliary cholangitis with an inadequate response to ursodeoxycholic acid: a double-blind, randomised, placebo-controlled, phase 2, proof-of-concept study. *Lancet Gastroenterol Hepatol* 2017. 2: 716–726.
